# Regioisomeric control of layered crystallinity in solution-processable organic semiconductors†

**DOI:** 10.1039/d0sc04461j

**Published:** 2020-10-19

**Authors:** Satoru Inoue, Toshiki Higashino, Shunto Arai, Reiji Kumai, Hiroyuki Matsui, Seiji Tsuzuki, Sachio Horiuchi, Tatsuo Hasegawa

**Affiliations:** Department of Applied Physics, The University of Tokyo 7-3-1 Hongo Tokyo 113 8656 Japan satoru.inoue@ap.t.u-tokyo.ac.jp t-hasegawa@ap.t.u-tokyo.ac.jp; Research Institute for Advanced Electronics and Photonics (RIAEP), National Institute of Advanced Industrial Science and Technology (AIST) Tsukuba Ibaraki 305-8565 Japan; Condensed Matter Research Centre (CMRC) and Photon Factory, Institute of Materials Structure Science, High Energy Accelerator Research Organization (KEK) Tsukuba Ibaraki 305-0801 Japan; Research Center for Organic Electronics, Yamagata University Yonezawa Yamagata 992-8510 Japan; Research Center for Computational Design of Advanced Functional Materials (CD-FMat), National Institute of Advanced Industrial Science and Technology (AIST) Tsukuba Ibaraki 305-8568 Japan

## Abstract

The construction and control of 2D layered molecular packing motifs with functionally substituted π-electron cores are crucial for developing organic electronic materials and devices. We investigated a regioisomeric structure–property relationship in high-performance and solution-processable layered organic semiconductors based on *mono*-octyl-substituted benzothieno[3,2-*b*]naphtho[2,3-*b*]thiophene (*mono*-C8-BTNT). We demonstrated that an isomorphous *bilayer-type* layered herringbone packing motif is obtainable in a series of four positional isomers of *mono*-C8-BTNTs whose π-electron core is substituted by an octyl chain at one of the four most peripheral positions with roughly keeping the rod-like molecular shape. These regioisomeric compounds exhibited systematic variations in the solvent solubility and liquid-crystalline phase transitions at elevated temperatures. The analysis of intermolecular interaction energies in the crystals based on dispersion-corrected DFT calculations revealed that the crystals of 2- and 8-*mono*-C8-BTNTs are more stable than those of 3- and 9-*mono*-C8-BTNTs owing to the higher ordering of alkyl chain layers in the crystals. Such differences of the stability in their crystal formation are closely correlated with TFT performances, where the single-crystal devices of the 2- and 8-*mono*-C8-BTNTs substituted at the most peripheral positions exhibit high-performance TFT characteristics with a mobility of approximately 10 cm^2^ V^−1^ s^−1^.

## Abstract 2

An isomorphous *bilayer-type* layered herringbone crystal packing is reported for a series of four positional isomers of *mono*-C8-BTNTs, where the single-crystal devices with the isomers exhibit high-performance TFT characteristics.

## Graphical abstract

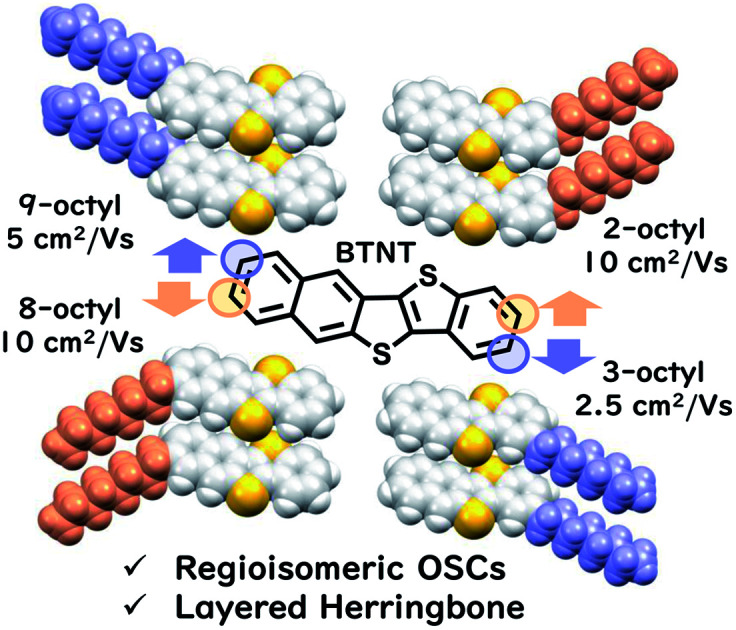

## Introduction

Solution-processable organic semiconductors (OSCs) are composed, in most cases, of π-electron cores bonded with functional substituents.^1–5^ The substituents are structurally flexible and electrically inert, as observed in typical substituents such as normal and branched alkyl chains,^6–15^ silylethynyl,^16–19^ fluoroalkyl,^20–24^ or others,^25–29^ which is in contrast to the rigid nature of π-electron cores that play major roles in the carrier transport characteristics. However, the role of the substituents is often critical, not only for increasing the solubility characteristics, but also for affecting the whole material characteristics through the variation of intermolecular packing geometries. The important issue is to identify optimal combinations between the π-electron cores and the substituents, including their substituting positions, to develop prolific organic electronic applications.

Among a variety of substituents, normal alkyl-chain substitutions are especially significant for achieving high-performance OSCs as used in thin-film transistors (TFTs).^30–44^ In particular, it has been demonstrated recently that self-organized alkyl-chain layers play essential roles in enhancing the layered crystallinity, which is effective and useful for the formation of perfectly aligned semiconductor–insulator interfaces in TFT device structures.^45–48^ For example, a simple alkyl-chain substitution on some π-electron cores, such as benzothieno[3,2-*b*]benzothiophene (BTBT), allows the formation of *bilayer-type* layered herringbone (*b*-LHB) packing motifs;^45–52^ the alkylated unsymmetric rod-like molecules form unipolarly aligned molecular layers by their side-by-side intermolecular arrangements. Consequently, the resultant unipolar layers form an alternating antiparallel stacking alignment such that the π-core layers and alkyl chain layers are in head-to-head (tail-to-tail) contact within the crystal. High-precision quantum chemical calculations for the intermolecular interaction energies revealed that the crystalline stability is considerably enforced due to the aligned alkyl-chain layer formation, which assists the π-core-layer formation.^46,53^ Also, it has been demonstrated recently that alkyl-chain substitution is effective in realizing the independent layer formation of π-cores in crystals, even though the unsubstituted π-cores do not exhibit layered molecular packing motifs.^53^

Despite these findings, however, the controllability or designability has not been investigated yet for layered molecular packing by functional substitutions to produce high-performance OSCs. In particular, the effect and role of the substituting positions on OSCs remain unclear, whereas such regioisomerism is generally known as a powerful methodology to modify and develop organic molecules, as demonstrated in broad research fields for fine chemicals^54–59^ and/or pharmaceuticals.^60–63^ Such an ambiguous nature can be attributed to the fact that the change of substituting positions may violate the layered crystallinity, which eventually prevents the uniform layer formation of OSCs and thus contributes to deteriorating the semiconducting properties of TFTs.^42,64–67^ Nonetheless, the systematic understanding of the regioisomeric effect has not yet been achieved in OSCs.

**Chart 1:**
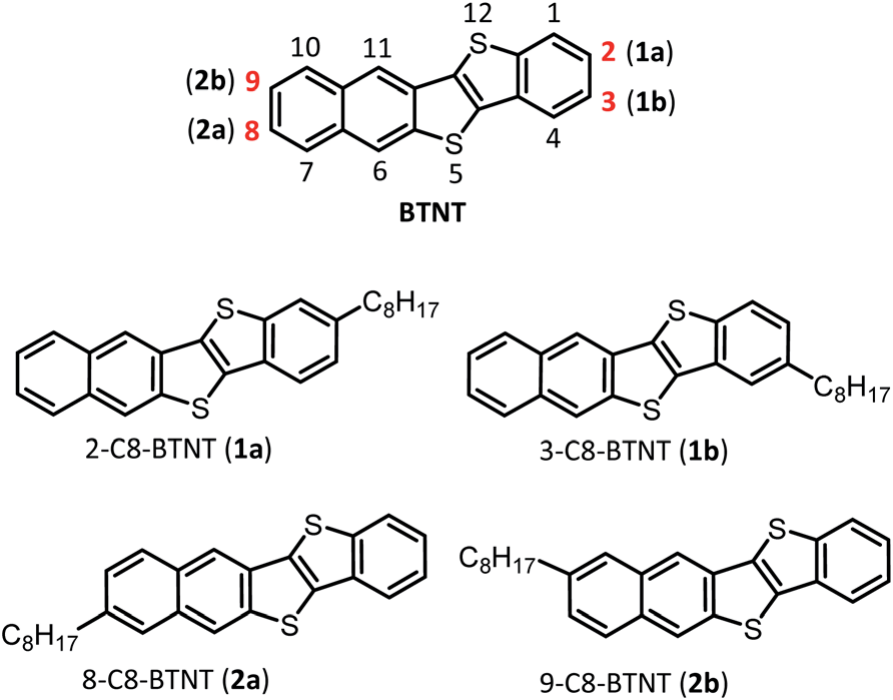
Chemical structures of four positional isomers of *mono*-C8-BTNTs.

In this study, we investigated the effect of substituting positions on the structure–property relationships of high-performance and solution-processable layered-crystalline OSCs, with a focus on an extended π-electron core of benzothieno[3,2-*b*]naphtho[2,3-*b*]thiophene (BTNT)^68^ substituted simply by a normal octyl chain, which is denoted as *mono*-C8-BTNT. As the BTNT core does not have inversion or mirror symmetry, we could choose four unique (or independent) substituting positions, with roughly keeping the rod-like molecular shape over the whole molecules, as presented in Chart 1; 1a (2-C8-BTNT) and 2a (8-C8-BTNT) were substituted at the two most peripheral positions of the rod-like π-core of BTNT, while 1b (3-C8-BTNT) and 2b (9-C8-BTNT) were substituted at the two second most peripheral positions. We found that all four regioisomers formed an isomorphous *b*-LHB molecular packing motif that is the most suitable for TFT device structure formation. We present and discuss the crystal structure–property relationship based on the regioisomers in terms of the thermal stability, solubility, and TFT device characteristics. We showed that the *b*-LHB molecular packing motif and the materials' characteristics undergo a systematic change by varying the substituting positions.

## Results and discussion

### Material synthesis

**Chart 2:**
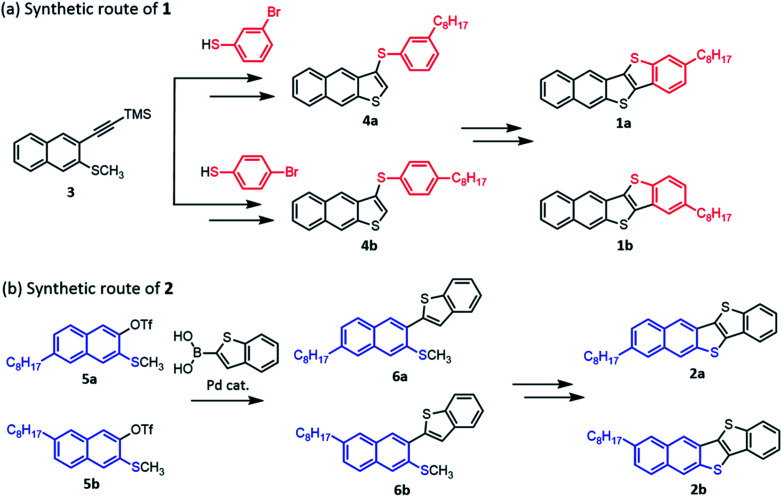
Two synthetic routes to *mono*-C8-BTNT regioisomers.

We examined two synthetic routes as illustrated in Chart 2 to obtain *mono*-C8-BTNT regioisomers selectively. 1a and 1b were synthesized according to a reported procedure of BTNT derivatives^49,68^ with several modifications. As shown in Chart 2(a), the key intermediates 4a and 4b for obtaining 1a and 1b were synthesized by a thiophene-annulation reaction between naphthalene derivative 3 and corresponding bromobenzenethiols, which is followed by palladium catalyzed cross-coupling with octylboronic ester. Subsequent iodization and palladium catalyzed intramolecular coupling afforded 1a and 1b. As shown in Chart 2(b), the key intermediates 6a and 6b for obtaining 2a and 2b were synthesized by the Suzuki coupling reaction between benzothiopheneboronic acid and corresponding triflates 5a and 5b. Subsequent oxidization and intramolecular cyclization with triflic acid afforded 2a and 2b. The details of all synthetic procedures are described in the ESI.† The final products were purified by column chromatography and repeated recrystallization from a mixed solution of chloroform and ethanol.

### Crystalline morphologies of regioisomers

**Fig. 1:**
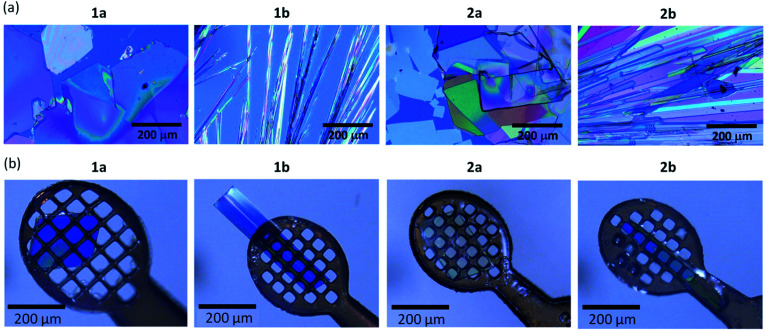
Micrographs for single crystals of *mono*-C8-BTNT regioisomers. (a) Precipitated crystals grown by drop-casting of anisole solution at room temperature. (b) Single crystals for crystal structure analyses, obtained by recrystallization from anisole (1a, 2a, and 2b), and from a mixed solution of chloroform and ethanol at room temperature (1b).

Crystalline morphologies of all the compounds were flake (plate) like, although the obtained crystal size depends on the respective compounds, as presented in Fig. 1. The crystals of 1a and 2a are slightly larger and are obtained as more plate-like, while those of 1b and 2b are more needle-like.

### Isomorphous packing and regioisomeric effects

**Fig. 2:**
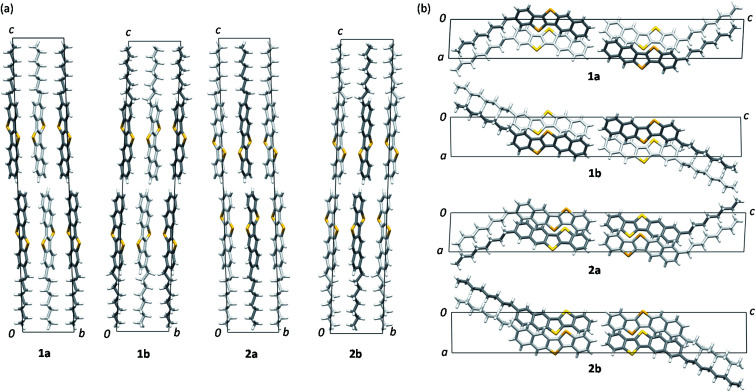
Unit-cell packing diagrams of *mono*-C8-BTNT regioisomers. (a) Projection of the *b*–*c* plane. (b) Projection of the *a*–*c* plane. Crystallographically independent molecules are shown, respectively, in different colors (white and gray). Sulfur atoms are shown in yellow color.

Views of crystal structures are shown in Fig. 2 for all four types of regioisomers of *mono*-C8-BTNTs. All the compounds exhibited an isomorphous *b*-LHB molecular packing motif with the same space group symmetry of *P*1̄. In the crystals, the BTNT cores formed head-to-head alignment between the unipolar layers, irrespective of the substituting positions. Thus, head-to-head contacts were observed between the naphthalene-ring sides of the BTNT cores in 1a and 1b, and between the benzene-ring sides in 2a and 2b. The details of interlayer molecular packings are shown in Fig. S1–S5.†

**Fig. 3:**
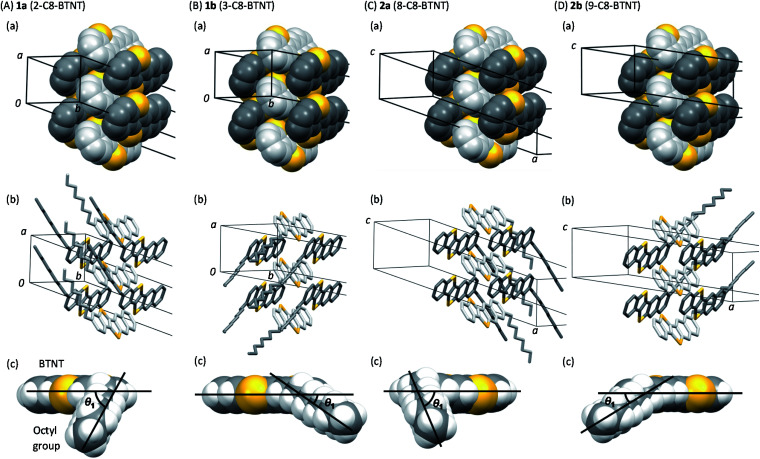
3D intermolecular arrangements of *mono*-C8-BTNT regioisomers. (a) Space-filling views for intralayer 3D packing of BTNT cores, and also shown by (b) stick models with alkyl chains. Each crystallographically independent molecule is shown in different colors (white and gray) in (a) and (b). (c) Space-filling views along both the BTNT core plane and the all-*trans* octyl chain plane. Definition of the dihedral angle (*θ*_1_) between the planes is also shown. Sulfur atoms are shown in yellow color.

Fig. 3 presents the space-filling views of the intermolecular arrangements between the BTNT cores for all the regioisomer crystals. It was found that the *b*-LHB arrangements between the BTNT cores were almost the same for all the regioisomers, despite the change in the substituting positions from which the alkyl chains were sprouted out in the crystals. Meanwhile, the substituted alkyl chains formed all-*trans* chain planes (see Fig. 3) arranged to form ordered alkyl-chain layers through the *T*-shaped and slipped parallel contacts between the chain planes. The study demonstrated that the characteristic angles formed by the core planes and chain planes present a systematic variation depending on the substituting positions, as presented in Table 1. All the inter-core herringbone angles *θ*_2_ were roughly the same for all the compounds. In contrast, the interchain herringbone angle *θ*_3_ and core-chain dihedral angle *θ*_1_ differed significantly between the two groups; those of 1a and 2a and those of 1b and 2b were similar to each other, respectively, but the former was much different from the latter. The differences between the two groups are associated with the feature of the substituting positions; those of 1a and 2a were located at the most peripheral positions, while those of 1b and 2b were located at the second most peripheral positions.

**Table 1:** Crystallographic parameters and values of the intramolecular dihedral angle between the BTNT core plane and all-*trans* chain plane (*θ*_1_), core–core herringbone angle (*θ*_2_) and inter-core distance (*d*_2_), chain–chain herringbone angle (*θ*_3_) and inter-chain distance (*d*_3_)

	Crystallographic parameters							Dihedral angles	Packing diagrams of BTNT cores		Alignment of octyl chains	
	*a* (Å)	*b* (Å)	*c* (Å)	*α* (deg)	*β* (deg)	*γ* (deg)	*V* (Å^3^)	*θ*_1_ (degree)	*θ*_2_ (degree)	*d*_2_ (Å)	*θ*_3_ (degree)	*d*_3_ (Å)
1a	6.01908 (11)	7.87883 (16)	45.2168 (12)	91.9646 (19)	92.9466 (18)	90.3409 (16)	2140.17 (8)	63.36	53.95	2.71	80.45	3.30
1b	6.0576 (3)	8.0009 (4)	44.519 (3)	87.629 (5)	88.944 (4)	89.750 (4)	2155.4 (2)	29.85	54.90	2.76	111.58	4.26
2a	6.00978 (14)	7.87052 (18)	45.2747 (12)	91.737 (2)	92.907 (2)	90.2170 (18)	2137.73 (9)	65.38	53.76	2.71	84.93	3.51
2b	6.1144 (3)	7.9480 (5)	44.660 (2)	87.923 (5)	89.367 (4)	89.762 (5)	2168.8 (2)	34.57	53.24	2.71	114.45	4.51

### Intermolecular interaction energies

**Fig. 4:**
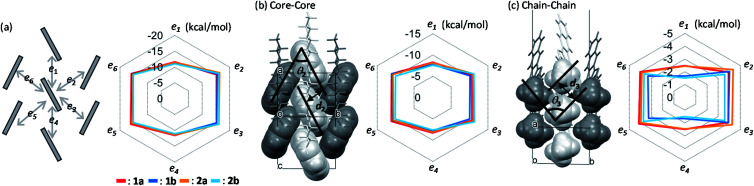
Calculated intermolecular interaction energies of *mono*-C8-BTNT regioisomers. (a) Total intermolecular interaction energies between adjacent molecules at the *T*-shaped (*e*_2_, *e*_3_, *e*_5_, and *e*_6_) and slipped parallel (*e*_1_ and *e*_4_) contacts. Scheme for the interlayer herringbone packing motif is shown on the left. (b) Contributions from the core–core interactions. Space-filling view for the intralayer core–core arrangement with the definition of the herringbone angle (*θ*_2_) and inter-core distance (*d*_2_) is also shown on the left. (c) Contributions from the chain–chain interactions. Space-filling view for the intralayer chain–chain arrangement with the definition of the herringbone angle (*θ*_3_) and inter-chain distance (*d*_3_) is also shown on the left.

The calculated intermolecular interaction energies between neighboring molecules in the crystals are summarized in Fig. 4. The interaction energies between π-electron cores are roughly the same regardless of the substituting position by the alkyl chains (Fig. 4b), while those between alkyl chains depend clearly on the substituting positions (Fig. 4c). Additionally, the size ratios of the total intermolecular interaction energies between the *T*-shaped and slipped parallel contacts were kept at approximately 3 : 2 for all the compounds, the features of which were also observed in many other LHB compounds.^46,53^ It is most probable that the size ratio should remain the same to stabilize the LHB packing motifs.

A distinctive difference in the interchain interaction energies was observed between the two groups, as shown in Fig. 4c; 1a and 2a show higher interaction energies along the slipped parallel contacts than 1b and 2b. The result indicates that the formation of alkyl chain layers is energetically more beneficial in 1a and 2a than in 1b and 2b. Total cohesive energies, *E*_total_, are summarized in Table 2. Because of the higher interchain ordering in 1a and 2a than in 1b and 2b, cohesive energy is larger in the former than the latter, despite the same *b*-LHB packing. Actually, the volume of 1a and 2a in crystal cells is smaller than that of 1b and 2b. The results also imply that the alkyl-chain layer formation has more degrees of freedom than the core layer formation, which is the reason why the isomorphous *b*-LHB packing is kept for all four regioisomeric compounds. The alkyl chains are known to form a number of polymorphs in terms of the interchain ordering.^69–71^

**Table 2:** Lattice energies calculated from the crystal structures of *mono*-C8-BTNT regioisomers

	Lattice energy per molecules (kcal mol^−1^)			
	From intermolecular interaction energy			From QE
	*E*_total_ a	*E*_core–core_ b	*E*_chain–chain_ c	*E*_total_
1a	−42.88	−30.02	−10.50	−43.89
1b	−42.19	−28.76	−8.96	−42.34
2a	−43.10	−29.95	−10.70	−44.31
2b	−42.29	−28.97	−8.85	−42.51

a Lattice energies estimated from calculated interaction energies with six neighboring molecules in crystals.

b Contributions of interactions between aromatic cores.

c Contributions of interactions between alkyl chains.

### Intermolecular transfer integral

**Fig. 5:**
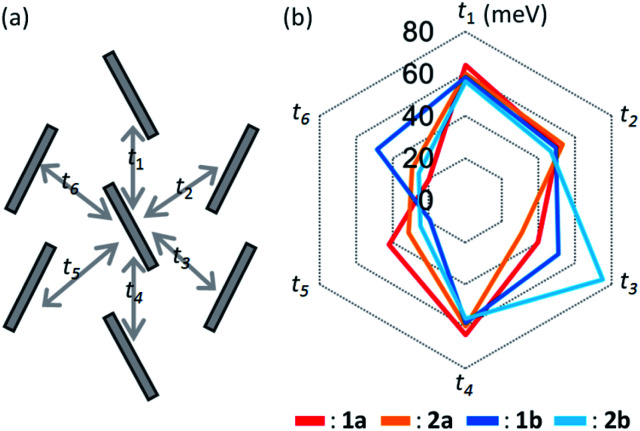
Calculated intermolecular transfer integrals of *mono*-C8-BTNT regioisomers. Scheme for the intralayer herringbone packing motif is shown on the left.

Fig. 5 shows the calculated transfer integrals. The values were estimated to be 60 meV between the slipped parallel contacts for all the regioisomers. In contrast, those between the *T*-shaped contacts varied between 20–80 meV, depending on the pairs of adjacent molecules, as well as on the different regioisomers. The feature is first ascribed to the unsymmetric nature of the BTNT core that does not have inversion or mirror symmetry, leading to nonequivalent relationships on the *T*-shaped contacts (*t*_2_, *t*_3_, *t*_5_, and *t*_6_) within the herringbone packing.

To investigate the origin of the above variation, we estimated the contributions of respective atomic pairs to the intermolecular transfer integrals, whose results are summarized in Fig. S6 and Table S2–S5.† Major contributions for both *T*-shaped and slipped parallel contacts are anti-phase overlaps between the atomic orbitals of sulfur and in-phase overlaps between the sp^2^-orbitals of carbon located around the center of the thienothiophene ring. It implies that the transfer integral is based on the sum of anti-phase and in-phase overlaps, whose balance is sensitive to the slight change of the packing motif. This feature makes us expect the discovery of OSCs, showing a much higher performance by chance in the near future.

### Solubility & thermal characteristics

**Fig. 6:**
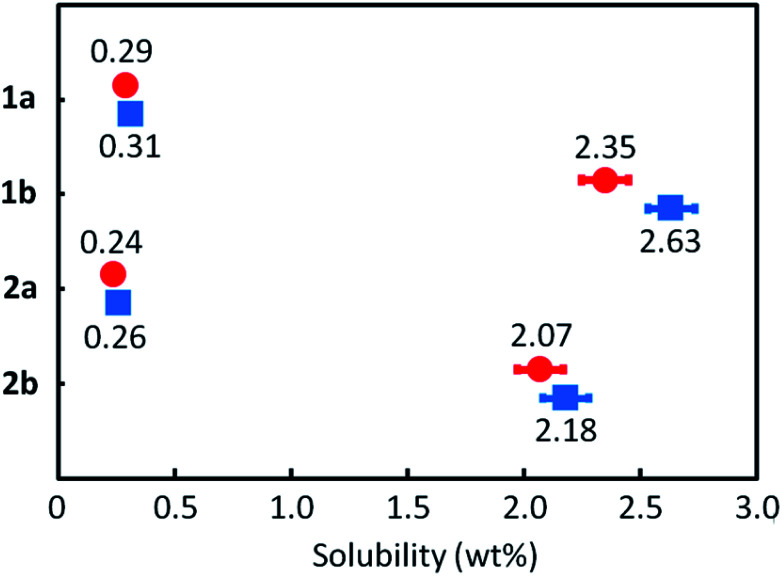
Solubility of *mono*-C8-BTNT regioisomers in *o*-xylene at 20 °C determined by the concentration of saturated solution. Red filled circles show the concentration estimated by the dissolution process, and blue filled squares show the concentration estimated by the precipitation process.

All the *mono*-C8-BTNT regioisomers were soluble in some aromatic solvents such as toluene, xylene, anisole, and tetralin. However, the solubility was lower in nonaromatic polar solvents such as *N*,*N*-dimethylformamide, acetonitrile, and ethanol. Fig. 6 shows their solubilities in *o*-xylene. The solubility of 1b (2.35 wt%) and 2b (2.07 wt%) is much higher than that of 1a (0.29 wt%) and 2a (0.24 wt%). The results are consistent with the calculated intermolecular interactions, which show that the crystals of 1a and 2a are more stable than those of 1b and 2b. We also found that the solubility estimated by the different processes (dissolution and precipitation) showed a slight difference in 1b and 2b, but not in 1a and 2a. We consider that the result also indicates the lower crystallinity of 1b and 2b as compared to 1a and 2a.

**Fig. 7:**
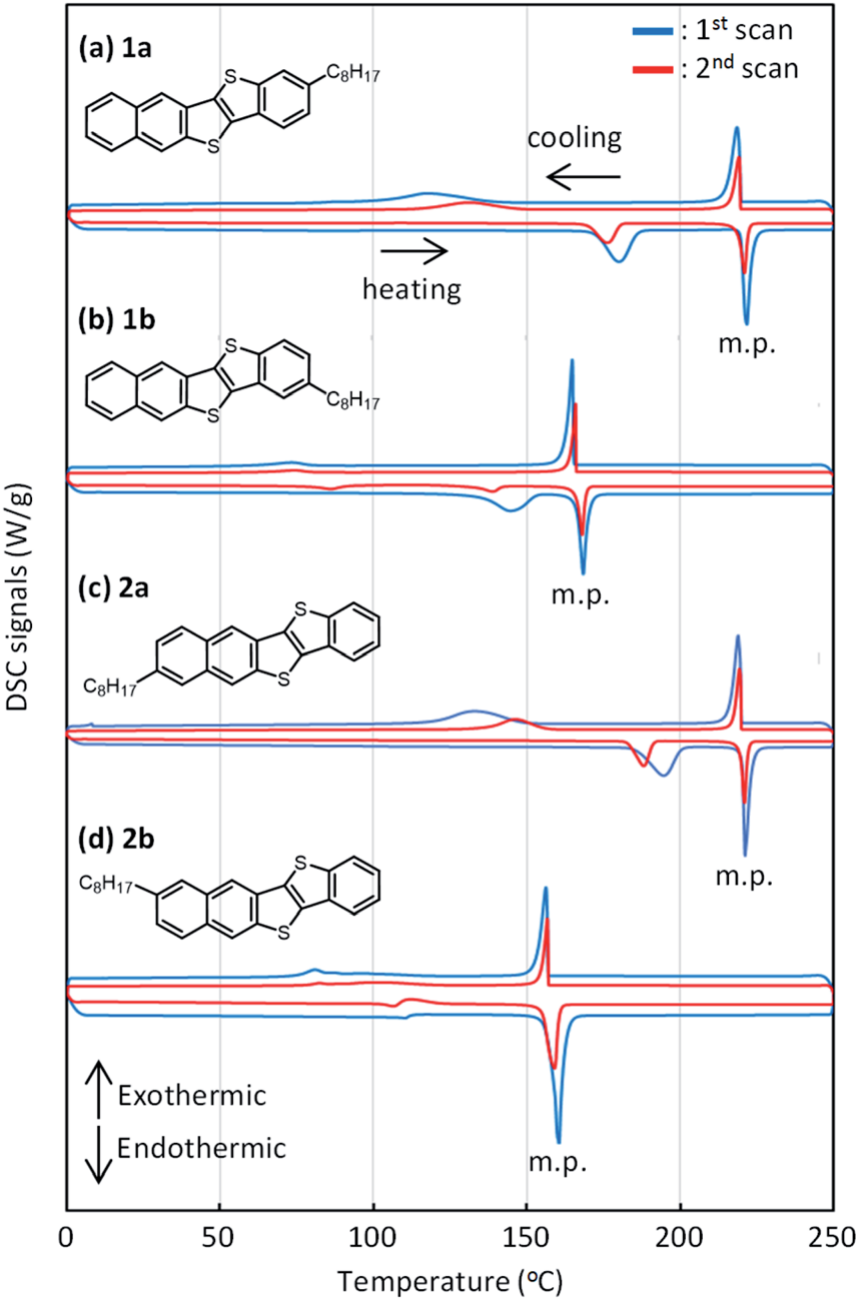
DSC curves of *mono*-C8-BTNT regioisomers. Blue curves show the results for scans at 10 K min^−1^, and red curves show the results for 2nd scans at 5 K min^−1^.

DSC charts for all the regioisomers are shown in Fig. 7. Both 1a and 2a exhibited two exothermic and endothermic peaks, respectively, associated with the liquid-crystal phase transition at 180–190 °C and the melting transition at 221–222 °C. Notably, the results indicate that the thermal durability of both 1a and 2a should be high enough for use in printed electronics. In contrast, both 1b and 2b showed similar but lower transition temperature (melting at 160–169 °C) than 1a and 2a. Fig. S7† shows the total enthalpies (Δ*H*_total_) estimated from the respective peaks of the DSC charts. We found that the Δ*H*_total_ values of 1a and 2a are approximately the same in the heating and cooling processes. In contrast, those of 1b and 2b estimated in the heating process are much larger than those estimated in the cooling process by *ca.* 7–10 kJ mol^−1^. We consider that 1b and 2b should show lower crystallinity or require a longer structural relaxation time for the crystallization, as compared to 1a and 2a.

We note here that two types of unsymmetrically substituted regioisomers were reported for Ph-BTNT-C10,^49^ in which the phenyl group and the alkyl chain were substituted at the most peripheral (2-alkyl-8-phenyl) and at the second most peripheral (3-alkyl-9-phenyl) positions. In the compounds, a similar trend was observed in the solubility and thermal characteristics, where the latter showed much higher solubility and lower melting points than the former.

### Polycrystalline thin films & TFT characteristics

**Fig. 8:**
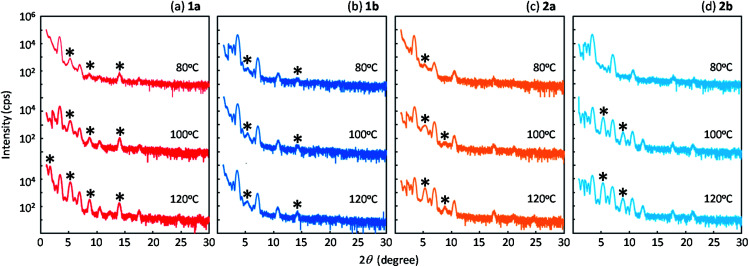
Out-of-plane X-ray diffraction profiles of spin-coated films of *mono*-C8-BTNT regioisomers with thermal treatment at 80 °C, 100 °C, and 120 °C for 5 min. Diffraction peaks due to the *d*-spacing of 4.5 nm, corresponding to the molecular bilayer thickness, are shown by the asterisk.

Fig. 8 shows the out-of-plane X-ray diffraction profiles of the spin-coated polycrystalline thin films treated by annealing at high temperature. We found that two types of diffraction peaks were observed for all the regioisomers, depending on the annealing conditions. The as-coated films and the films annealed at 80 °C exhibited distinctive diffraction peaks due to the *d*-spacing of 2.3 nm, which is approximately half of the lattice constant *c* (or molecular bilayer thickness), as shown in Table 1. By annealing at elevated temperatures, diffraction peaks due to the *d*-spacing of 4.5 nm, corresponding to the molecular bilayer thickness, appeared and grew, as shown by the asterisks. The *d*-spacing of 2.3 nm indicates the formation of a monomolecular layer, which should be grown by rapid solidification by the spin-coating process. The growth of diffraction peaks due to annealing was most clearly observed in the films of 1a. It is likely that the structural relaxation from the initial quenched phase to the stable *b*-LHB packing phase was promoted by thermal annealing conditions, depending on the thermal characteristics of each regioisomer. Similar annealing effects were also observed in polycrystalline thin films of Ph-BTBT-C10 and Ph-BTNT-C10.^49^ We consider that the rapid formation of the monomolecular layer and the structural relaxation of molecular bilayer films at elevated temperatures should be the common characteristics for the molecular bilayer organic semiconductors.

**Fig. 9:**
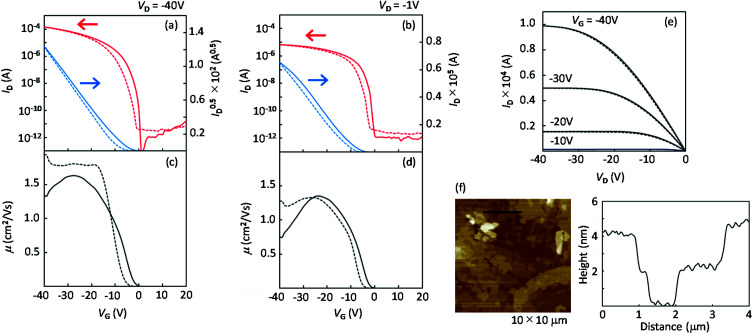
Typical device characteristics of spin-coated polycrystalline TFTs of 1a. (a) Transfer characteristics in the saturation regime and (b) in the linear regime. (c) and (d) show the plots for mobility as a function of *V*_G_. (e) Output characteristics. Solid lines and dashed lines are forward and backward scans, respectively. (f) AFM image for the spin-coated polycrystalline-thin film of 1a after annealing at 120 °C for 5 min.

**Fig. 10:**
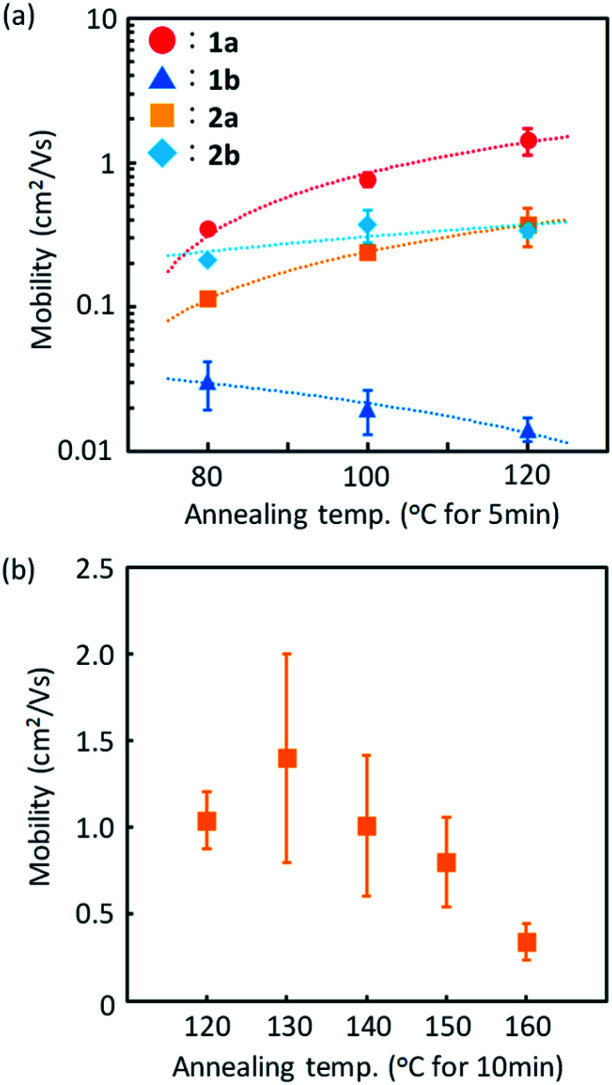
Effect of thermal treatment on the TFT mobility of spin-coated films of 1a (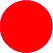), 1b (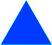), 2a (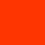), and 2b (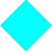) measured at a *V*_D_ of −40 V. (a) Changes in TFT mobilities for the four *mono*-C8-BTNT regioisomers. (b) Changes in TFT mobilities for 2a.

Fig. 9 presents the device characteristics of TFTs based on spin-coated polycrystalline films of 1a (the results for the other regioisomers are summarized in Fig. S8 and S9†). The field-effect mobility for both 1a and 2a was estimated to be more than 1 cm^2^ V^−1^ s^−1^ with low hysteresis and low turn-on voltage by annealing at elevated temperatures, although the measurements were conducted under ambient conditions. Table 3 summarizes the estimated values of polycrystalline TFT characteristics for all the regioisomers. Fig. 10 also show the dependence of TFT characteristics on the annealing temperature. We found that an improvement of the device mobility was observed in 1a and 2a by annealing at elevated temperature, but not in 1b and 2b. The results indicate the high thermal durability of 1a and 2a for practical use. In contrast, the mobility for 1b decreased by the increase of the annealing temperature. The result is consistent with the fact that the thin-film diffraction peaks, due to the *b*-LHB packing, were not observed in 1b. It is most probable that the quenching of the polycrystalline thin film from the initial drying state to the *b*-LHB crystalline state did not proceed effectively by the annealing treatment at temperatures higher than 80 °C for 1b. We here note that these mobility values are affected not only by the inclusion of quenched phases but also by the grain boundaries in polycrystalline thin films.

**Table 3:** Parameters of TFT characteristics after thermal treatment for spin-coated polycrystalline thin films of *mono*-C8-BTNT regioisomers. All the values were obtained as an average from more than 6 devices

	Annealing temp. (°C)	Annealing time (min)	Mobility (cm^2^ V^−1^ s^−1^)/standard deviation (%)		*V*_th_ (V)		*I*_on_/*I*_off_	
			*V*_D_ = −40 V	*V*_D_ = −1 V	*V*_D_ = −40 V	*V*_D_ = −1 V	*V*_D_ = −40 V	*V*_D_ = −1 V
1a	80	5	0.39/24	0.38/26	0.2 ± 3.9	−2.9 ± 3.2	10^6^	10^6^
	100		0.77/11	0.72/9	−4.9 ± 1.5	−8.2 ± 1.0	10^6^	10^7^
	120		1.43/21	1.26/20	−10.7 ± 3.5	−11.5 ± 2.8	10^7^	10^7^
1b	80	5	3.1 × 10^−2^/37	2.1 × 10^−2^/54	−1.1 ± 0.6	−10.4 ± 1.2	10^5^	10^5^
	100		2.0 × 10^−2^/34	1.0 × 10^−3^/20	−6.6 ± 5.0	−13.7 ± 0.9	10^5^	10^4^
	120		1.4 × 10^−2^/19	9.1 × 10^−3^/28	−2.2 ± 0.4	−9.7 ± 0.6	10^5^	10^4^
2a	80	5	0.11/9	0.11/15	−2.6 ± 0.4	−5.3 ± 0.6	10^5^	10^5^
	100		0.24/11	0.22/9	−3.5 ± 0.6	−6.1 ± 0.5	10^6^	10^5^
	120	5	0.37/29	0.30/25	−4.2 ± 2.0	−6.8 ± 1.3	10^6^	10^6^
		10	1.04/16	0.67/8	−12.6 ± 1.3	−13.7 ± 2.6	10^6^	10^6^
		20	1.54/11	1.02/5	−9.7 ± 1.7	−8.1 ± 1.4	10^6^	10^6^
2b	80	5	0.21/6	0.13/18	−0.8 ± 2.9	−6.1 ± 0.8	10^6^	10^6^
	100		0.37/25	0.33/22	−9.0 ± 2.6	−12.2 ± 1.3	10^6^	10^6^
	120		0.34/10	0.33/18	−9.2 ± 1.3	−12.4 ± 0.5	10^7^	10^6^

### Single-crystalline thin films & intrinsic mobility

In order to compare the intrinsic device performance between the regioisomeric compounds, it is necessary to use single-crystalline thin-film devices. By using very slow blade-coating, we successfully obtained crystalline thin films with large single-crystal domains for all the regioisomers. Optical microscope images and AFM profiles for all the films are shown in Fig. S9 and S10,† respectively. It was feasible to obtain large single-crystal domains in 1a and 2a, while it was much more difficult in 1b and 2b. As a result, the obtained domain size of 1b and 2b was limited and much smaller than that of 1a and 2a. It is clear that such a difference in the domain size should be associated with the degree of layered crystallinity in each regioisomers; 1a and 2a are more effectively promoted to have higher layered crystallinity by the alkyl chains than 1b and 2b. Note that the inter-π-core arrangements responsible for the carrier transport are retained in the single crystals of all the regioisomers, as discussed in the former section.

**Fig. 11:**
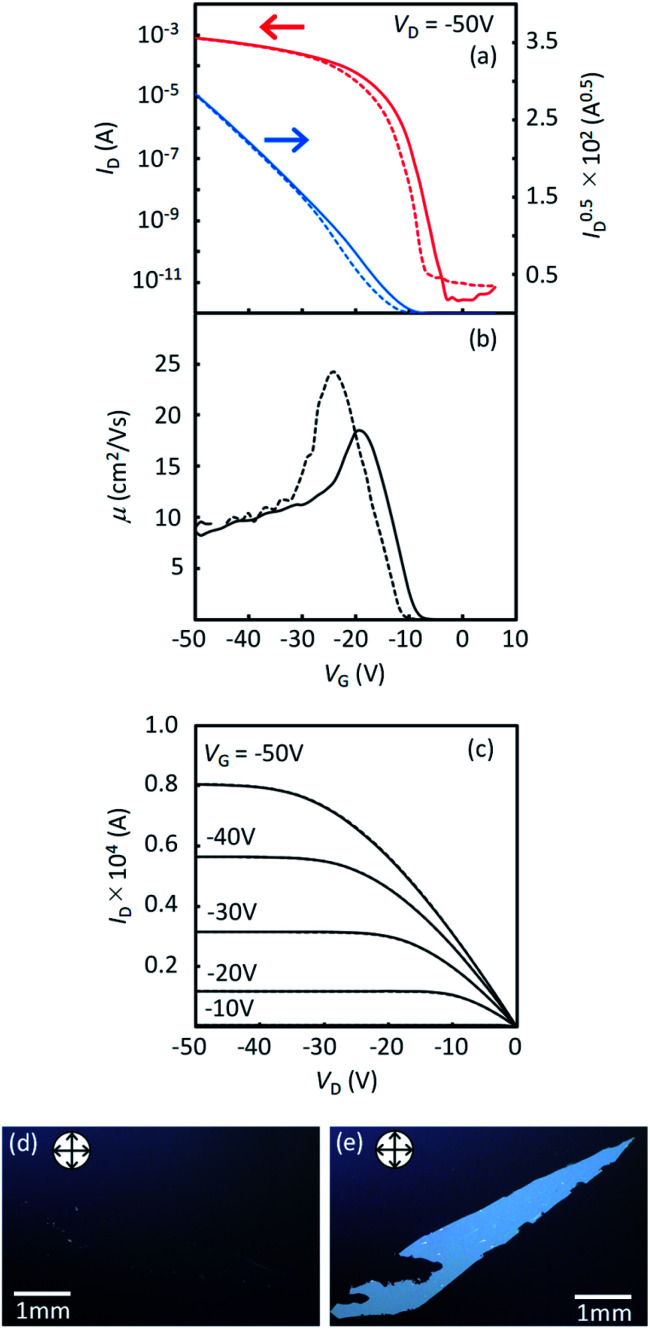
Device characteristics of single-crystal TFTs with 1a. (a) Transfer characteristics. (b) Plot of mobility as a function of *V*_G_. (c) Output characteristics. (d), (e) Crossed-Nicols polarized micrographs of a single-crystal thin film of 1a.

The typical transfer and output characteristics of single-crystal TFTs are shown in Fig. 11 for 1a, and in Fig. S11† for all the regioisomers. The device performance of the single-crystal TFTs are much higher than that of polycrystalline TFTs for all the *mono*-C8-BTNT regioisomers. Table 4 summarizes the estimated mobility, threshold voltage, and on/off current ratio. The intrinsic field-effect mobility of 1a and 2a in the saturation regime reaches as high as approximately 10 cm^2^/Vs. In contrast, 1b and 2b show relatively lower mobility at approximately 2.5 cm^2^ V^−1^ s^−1^ and 5 cm^2^ V^−1^ s^−1^, respectively, showing a large negative threshold voltage. These results imply that single-crystal thin films of 1b and 2b should involve a larger number of deep trap sites for carriers, associated with the lower degree of layered crystallinity, as stated above. We conclude that the closer *b*-LHB packing achieved by the substitution at the most peripheral positions is more favorable both for the crystalline stability and the semiconducting properties of *mono*-alkylated BTNTs.

**Table 4:** Parameters of TFT characteristics for single crystal thin films of *mono*-C8-BTNT regioisomers

VD (V)	Mobility (cm^2^ V^−1^ s^−1^)		*V*_th_ (V)		*I*_on_/*I*_off_	
	−50	−5	−50	−5	−50	−5
1a	10.3	8.3	−9.6	−12.7	10^8^	10^7^
1b	2.5	1.8	−26.2	−27.1	10^7^	10^7^
2a	10.3	2.8	−6.0	−4.7	10^7^	10^6^
2b	5.1	3.4	−35.5	−38.1	10^7^	10^7^

## Conclusions

We investigated the regioisomeric structure–property relationship in layered organic semiconductors based on alkyl-substituted BTNTs. We found that a series of four positional isomers whose BTNT core is substituted by the octyl chain at either of the four most peripheral positions afford isomorphous *b*-LHB molecular packing. The intralayer herringbone arrangements of the BTNT cores were almost the same for all the regioisomers, while the all-*trans* chain planes of the substituents formed two groups of interchain ordering depending on the substituting positions within the crystals: 1a and 2a, substituted at the most peripheral positions, and 1b and 2b, substituted at the second most peripheral positions. The thermal and solubility characteristics were determined by the difference in crystalline stability between the groups of these regioisomers. The results were consistent with the calculated intermolecular interaction energies in the crystals, where the crystals of 1a and 2a, substituted at the most peripheral positions, are more stable than those of 1b and 2b. It was also found that the higher crystalline stability of 1a and 2a is more favorable for providing larger single-crystal domains as well as higher semiconducting properties. Especially, 1a and 2a exhibit higher intrinsic field-effect mobility as high as approximately 10 cm^2^ V^−1^ s^−1^. The results indicate that an additional factor of “crystallinity”, which remains chemically unexplored, is crucial for the further development of organic semiconductors. These findings also demonstrate that the regioisomeric control allows the design of practical layered organic semiconductor materials with sufficient solvent solubility and thermal stability in addition to excellent carrier transport characteristics through the enhancement of the layered crystallinity. We envision that these findings should be also applicable for various aspects of molecular engineering in broad fields of chemical science.

## Experimental

### Structural analysis

Single crystals for structural analyses were obtained by recrystallization from saturated solution in anisole at room temperature for 1a, 2a, and 2b. Those for 1b were obtained by recrystallization from a solution in a mixed solvent of chloroform and ethanol through solvent evaporation at room temperature. The obtained crystals were carefully mounted on mounting apparatuses for structural analyses (LithoLoops, Molecular Dimensions Ltd.). Single-crystal X-ray diffraction measurements were conducted by using a Rigaku AFC10 four-circle diffractometer equipped with a Pilatus 200 K hybrid pixel detector for 2a, and a Rigaku VariMax Dual four-circle diffractometer equipped with a Pilatus 200 K for 1a, 1b, and 2b. All the structural analysis calculations were conducted using the CrysAlisPro software package^72^ and Crystal-Structure software package^73^ (Rigaku Co., Ltd.). The structures were solved by a direct method using the SIR92 (ref. 74) and the SIR2004 program.^75^ In the analyses, sulfur and carbon atoms were refined anisotropically, while hydrogen atoms were refined with the riding model using SHELXL97.^76^ All the crystallographic parameters are listed in Table S1.†

### Theoretical calculation

Density functional theory (DFT) calculations for the intermolecular interaction energies between adjacent molecules were performed using the Gaussian 16 program package,^77^ based on the molecular packing geometries as obtained by the crystal structure analyses. The intermolecular interaction energies were calculated at the B3LYP/6-311G** level with Grimme's D3 dispersion correction.^78,79^ The basis set superposition error (BSSE)^80^ was corrected by the counterpoise method.^81^ The interaction energies between BTNT cores and those between alkyl chains were calculated, respectively, using the component structures fragmented in the crystals. The dangling bonds formed by the fragmentations were capped by hydrogen atoms in the calculations. The contributions of the interactions with six neighboring molecules to the lattice energy per molecule (*E*) was calculated as half the sum of the interaction energies (*E*_i_) with the neighboring molecules as:
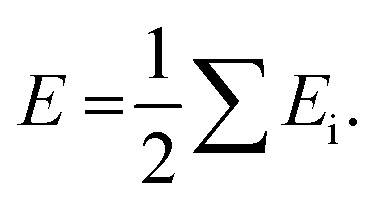


The lattice energy was also calculated using another electronic-structure calculation package of Quantum Espresso (QE),^82,83^ in which the crystal structures with a unit cell composed of four molecules were locally optimized at the PBE^84^ level with Grimme's D3 dispersion correction. The lattice energies *E* estimated by the QE were obtained by using the following equation:^85^*E* = *E*_cryst_ − 4*E*_mono_,where *E*_cryst_ is the energy of crystal structures with a unit cell composed of four molecules, and *E*_mono_ is the energy of the isolated molecule.

Intermolecular electron transfer integrals were also calculated by DFT calculations using the Gaussian 09 program package^86^ at the B3LYP/6-31G* level, based on the molecular packing geometries as obtained by the crystal structure analyses. The transfer integral *t*_*AB*_ between the molecular orbitals |*A*〉 and |*B*〉 was calculated by using the following equation:^87^
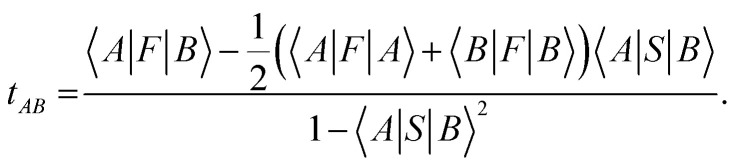
where *F* is the Fock matrix, and *S* is the overlap matrix. Considering that the molecular orbitals can be represented as the linear combination of atomic orbitals |*i*〉,|*j*〉 as:
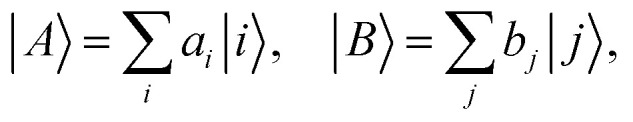
the transfer integral *t*_*AB*_ was decomposed into the contributions of all atomic orbital pairs *t*_*ij*_ as:
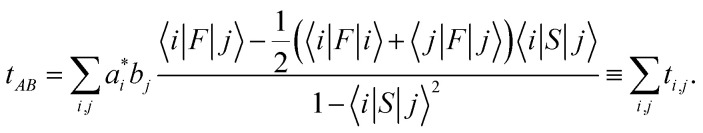


### Thermal characteristics

The powdered samples were thermally analyzed by differential scanning calorimetry (DSC; DSC7000X, Hitachi High-Tech Science Co.) at a scan rate of 5–10 K min^−1^. The measured temperature was calibrated using the melting point of indium (429.8 K), and synthetic sapphire was used as the standard for determining the heat capacity. For the DSC measurements, the powdered sample was heated and subsequently cooled at rates of 10 K min^−1^ (first scan) and 5 K min^−1^ (second scan). The melting points of all the compounds were determined by visual inspection of the changes in powdered samples on a hot plate. Transition enthalpies (Δ*H*) were estimated by the integration of respective DSC peaks. All the results are summarized in Tables S6 and S7.†

### Solubility

The solubility of the respective materials was estimated by the following two methods. The first one is the estimation of the solubility by inspecting the dissolution process at room temperature. Organic solvents were added to a powdered sample (3–5 mg) in increments of 20 μL using a micropipette, and the mixture was stirred to dissolve the sample completely at 20 °C. The complete dissolutions in organic solvents were confirmed by visual inspection. The second method is the estimation of the solubility by inspecting the precipitation process with decreasing the temperature: a number of solution vials with different concentrations were prepared at 80 °C, and subsequently cooled down to 20 °C with continuous stirring for 12 h. The solubility was calculated from the sample weight and the total volume of the solvent.

### Thin-film fabrication and characterization

We prepared two types of thin films of *mono*-C8-BTNT; polycrystalline thin films were fabricated by spin-coating, and single-crystal thin films were fabricated by blade coating. In the fabrication of all the spin-coated films, 0.4 wt% semiconductor solutions in *o*-xylene were used. The solution membranes were obtained by short-term spin-coating (1000 rpm/3 s), and subsequently annealed to produce polycrystalline semiconductor layers by the post-evaporation of solvents. The thickness of the film was *ca.* 15–20 nm, as was estimated by AFM measurements. In the fabrication of single-crystal films, 0.1 wt% semiconductor solutions in chlorobenzene for 1a, 2a, and 2b and 0.15 wt% of anisole for 1b were used for blade-coating at sweep rates of 3.0 μm s^−1^ for 1a and 2a and 1.5 μm s^−1^ for 1b and 2b at room temperature (23 °C). Optical microscope images were collected by using a digital microscope (VHX-6000; Keyence Co., Ltd.).

The measurements of out-of-plane X-ray diffraction profiles were carried out with a thin-film diffractometer (SmartLab; Rigaku Co., Ltd.) by using a monochromatized synchrotron radiated X-ray beam with an energy of 9.0 keV at the beamlines BL-7C of Photon Factory (PF) in KEK. Diffraction intensity was recorded using a scintillation counter. The film thickness and height profiles of the films were measured by tapping mode AFM (VN-8010; Keyence Co., Ltd. and Cypher; Asylum Research Inc., USA).

### Device fabrication and characterization

Two types of organic TFTs based on the *mono*-C8-BTNT thin film were fabricated; one was bottom-gate, top-contact TFTs composed of spin-coated polycrystalline thin films, and the other was bottom-gate, top-contact TFTs composed of blade-coated single-crystal thin films. We used highly doped (p^+^)-Si wafers with 100 nm-thick silicon dioxide layers as substrates for all the devices. Source/drain electrodes were fabricated by vacuum deposition of Au through a shadow mask to define the length (*L*) and width (*W*) of all the TFT channels, respectively, at 100 and 500 μm for polycrystalline thin films, at 200 and 500 μm for single-crystal thin films of 1a and 2a, and at 100 and 300 μm for single-crystal thin films of 1b and 2b. A micromanipulator (AxisPro; Systems Engineering Inc.) was used to trim away the films outside the channels for the proper evaluation of the device mobility.

The OTFT characteristics were measured using a semiconductor parametric analyzer (E5270A; Agilent Technologies Co. Ltd., B2912A; Keysight Technologies Inc.) under ambient conditions. The field-effect mobility (*μ*) was defined as the derivative of the transfer curve according to the following equations:
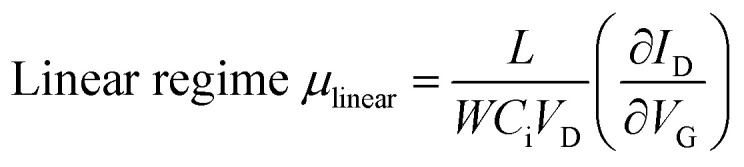

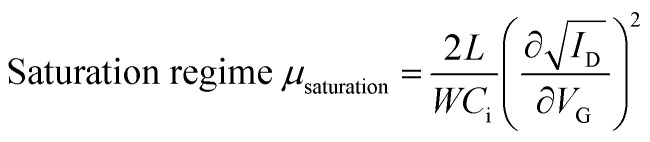
where *I*_D_, *C*_i_, *V*_D_, and *V*_G_ are the drain current, gate capacitance per unit area, drain voltage, and gate voltage, respectively. The measurements were conducted at a *V*_D_ of −1 V in the linear regime and at a *V*_D_ of −40 V in the saturation regime for polycrystalline TFTs and at a *V*_D_ of −5 V in the linear regime and at a *V*_D_ of −50 V in the saturation regime for single-crystal TFTs.

## Conflicts of interest

There are no conflicts to declare.

## Supplementary Material

SC-011-D0SC04461J-s001

SC-011-D0SC04461J-s002
