# Supplementary material for: Regioisomeric control of layered crystallinity in solution-processable organic semiconductors
Source: Chem Sci. 2020 Oct 19;11(46):12493–505. doi: 10.1039/d0sc04461j (PMC8647348; doi:10.1039/d0sc04461j)
Supplement: SC-011-D0SC04461J-s001 [file SC-011-D0SC04461J-s001.pdf]

Electronic Supplementary Material (ESI) for Chemical Science.
This journal is © The Royal Society of Chemistry 2020

Supporting Information

Regioisomeric Control of Solution-Processable Layered Organic Semiconductor Crystals

Satoru Inoue,[*,†] Toshiki Higashino,[‡] Shunto Arai,[†] Reiji Kumai,[§] Hiroyuki Matsui,[#] Seiji Tsuzuki,[‖]

Sachio Horiuchi,[‡] Tatsuo Hasegawa[*,†]

[†]Department of Applied Physics, The University of Tokyo, 7-3-1 Hongo, Tokyo 113 8656, Japan

[‡]Research Institute for Advanced Electronics and Photonics (RIAEP), National Institute of Advanced Industrial Science and Technology (AIST), Tsukuba, 305-8565, Japan

[§]Condensed Matter Research Centre (CMRC) and Photon Factory, Institute of Materials Structure Science, High Energy Accelerator Research Organization (KEK), Tsukuba 305-0801, Japan

[#]Research Center for Organic Electronics, Yamagata University, Yonezawa, Yamagata 992-8510, Japan

‖Research Center for Computational Design of Advanced Functional Materials (CD-FMat), National Institute of Advanced Industrial Science and Technology (AIST), Tsukuba 305-8568, Japan

Contents

1. **Materials Synthesis** S2
2. **Structural Characteristics** S12
3. **Intermolecular Transfer Integrals** S16
4. **Thermal Characteristics** S21
5. **Device Characterization** S23
6. **References** S26

1. Materials Synthesis

General Information

All chemicals and solvent are of reagent grade unless otherwise indicated. Nuclear magnetic resonance (NMR) spectra were recorded on a Bruker Avance500 spectrometer at 500MHz. Chemical shifts (d) are reported in ppm relative to tetramethylsilane (δ 0.00). The data of multiplicity are shown as follows: s = singlet, d = doublet, t = triplet, q = qualtet, m = multiplet. Mass analyses (MS) were performed on a GC-MS PQ2010SE in an electron impact ionization procedure.

Synthesis of 2-C8-BTNT (1a)

Scheme S1 shows synthetic route of **2-C8-BTNT (1a)**, which was carried out by the combination of reported procedure [S1-S5] with several modifications.

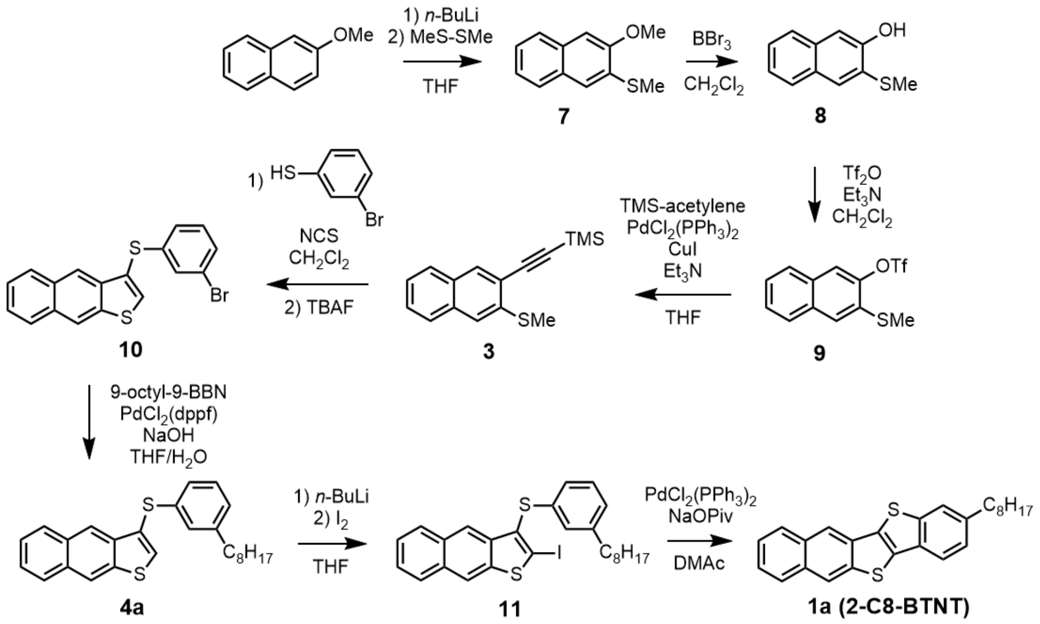

Scheme S1. Synthetic route of **2-C8-BTNT (1a).**

2-Methoxy-3-methylthionaphthalene (7)[S1]**.** To a solution of 2-methoxynaphthalene (10.0 g, 63.2 mmol) in THF (150 mL) cooled with ice bath was added dropwise n-BuLi (1.6 M hexane solution, 51.4 mL, 82.2 mmol, 1.3 eq) under N_2 atmosphere. After the mixture was stirred for 1 h keeping with ice-bath condition, dimethyldisulfide (7.74 g, 82.2 mmol, 1.3 eq) was added slowly to the solution. The resulting mixture was warmed to room temperature and then stirred for 1 h. The mixture was poured into water and extracted with chloroform. The combined organic layer was separated and concentrated under reduce pressure. The

resulting solid was recrystallized from a methanol to afford **7** as a white crystal (9.60 g, 74%).
^{1}H NMR (500 MHz, $CDCl_3$): δ (ppm) 7.70 (d, J = 8.5 Hz, 1H), 7.70 (d, J = 8.5 Hz, 1H), 7.47 (s, 1H), 7.38 (dd, J = 8.5 Hz, 8.5 Hz, 1H), 7.33 (dd, J = 8.5 Hz, 8.5 Hz, 1H), 7.09 (s, 1H), 4.00 (s, 3H), 2.54 (s, 3H). MS (EI mode) m/z 204.

3-Methylthio-2-naphthol (8)[S1]**.** To a solution of **7** (9.6 g, 47.0 mmol) in dichloromethane (150 mL) cooled with ice bath was added dropwise BBr_3 (1.0 M dichloromethane solution, 61.1 mL, 61.1 mmol, 1.3 eq). After the mixture was warmed to room temperature and then stirred for 1h. The mixture was poured into water and extracted with chloroform. The combined organic layer was separated and concentrated under reduce pressure. The resulting crude product was passed through a silica gel pad with hexane-chloroform (5:1 v/v) as eluent to give **8** (8.8 g, 98 %) as a white solid.
^{1}H NMR (500 MHz, $CDCl_3$): δ (ppm) 8.01 (s, 1H), 7.72 (d, J = 8.5 Hz, 1H), 7.68 (d, J = 8.5 Hz, 1H), 7.42 (dd, J = 8.5 Hz, 8.5 Hz, 1H), 7.32 (dd, J = 8.5 Hz, 8.5 Hz, 1H), 7.32 (s, 1H), 6.61 (s, 1H), 2.43 (s, 3H). MS (EI mode) m/z 204.

3-Methylthionaphthalen-2-yl trifluoromethanesulfonate (9)[S1]**.** To a solution of **8** (9.30 g, 48.9 mmol) and triethylamine (5.94 g, 58.7 mmol, 1.2 eq) in dichloromethane (100 mL) cooled with ice bath was added dropwise trifluoromethanesulfonic anhydride (16.6 g, 58.7 mmol, 1.2 eq). After the mixture was warmed to room temperature and then stirred for 1h. The mixture was poured into water and extracted with chloroform. The combined organic layer was separated and concentrated under reduce pressure. The resulting crude product was passed through a silica gel pad with hexane as eluent to give **9** (13.6 g, 86 %) as a white solid.
^{1}H NMR (500 MHz, $CDCl_3$): δ (ppm) 7.80 (d, J = 8.0 Hz, 1H), 7.79 (d, J = 8.0 Hz, 1H), 7.73 (s, 1H), 7.69 (s, 1H), 7.54 (dd, J = 8.0 Hz, 8.0 Hz, 1H), 7.49 (dd, J = 8.0 Hz, 8.0 Hz, 1H), 2.59 (s, 3H). MS (EI mode) m/z 322.

2-Methylthio-3-[2-(trimethylsilyl)ethynyl] naphthalene (3)[S2,S3]**.** A mixture of **9** (10.0 g, 31.0 mmol), trimethylsilylacetylene (4.57 g, 46.5 mmol, 1.5 eq), triethylamine (15.7 g, 155 mmol, 5.0 eq), CuI (177 mg, 0.93 mmol, 3 mol%), and $PdCl_2(PPh_3)_2$ (1.09 g, 1.55 mmol, 5 mol%) in toluene (100 mL) was stirred for 6 h at 80 °C. The reaction mixture was poured into water and extracted with toluene. The combined organic layer was separated and concentrated under reduce pressure. The resulting crude product was passed through a silica gel pad with hexane as eluent to give **3** (7.57 g, 90 %) as orange oil.

^{1}H NMR (500 MHz, $CDCl_3$): δ (ppm) 7.96 (s, 1H), 7.72 (m, 2H), 7.45 (s, 1H), 7.45 (m, 1H), 7.39 (m, 1H), 2.58 (s, 3H), 0.31 (s, 9H). MS (EI mode) m/z 270.

3-[(3-Bromophenyl)thio]naphtho[2,3-*b*]thiophene (10). To a solution of 3-bromothiophene (5.2 g, 27.8 mmol) in dichloromethane (100 mL) cooled with ice bath was added N-chlorosuccinimide (3.7 g, 27.8 mmol). After the mixture was stirred for 1 h keeping with ice-bath condition, the resulting dichloromethane solution of 3-bromophenylsulfenyl chloride was added to a solution of **3** (5.0 g, 18.5 mmol) in dichloromethane (50 mL). After stirring at room temperature for 2 h, a solution of tetrabutylammonium fluoride (1.0 M, 25.0 mL, 25.0 mmol) was added to the mixture, and then stirred for 2 h. The reaction mixture was poured into water and extracted with chloroform. The combined organic layer was separated and concentrated under reduce pressure. The resulting crude product was purified by silica gel column chromatography (chloroform:hexane = 1:9) to afford **10** (6.1 g, 89 %) as yellow oil.
^{1}H NMR (500 MHz, $CDCl_3$): δ (ppm) 8.40 (s, 1H), 8.29 (s, 1H), 7.94 (d, J = 8.5 Hz, 1H), 7.93 (d, J = 8.5 Hz, 1H), 7.86 (s, 1H), 7.51 (dd, J = 8.5 Hz, 8.5 Hz, 1H), 7.47 (dd, J = 8.5 Hz, 8.5 Hz, 1H), 7.35 (d, J = 8.0 Hz, 1H), 7.18 (dd, J = 8.0 Hz, 8.0 Hz, 1H), 7.05 (m, 2H). MS (EI mode) m/z 370.

3-[(3-*n*-Octylphenyl)thio]naphtho[2,3-*b*]thiophene (4a). To a solution of 9-borabicyclo[3.3.1]nonane (9-BBN) in THF (0.5M, 49.3 mL, 24.6 mmol) was added dropwise 1-octene (2.76 g, 24.6 mmol) at room temperature under N_2 atmosphere. After stirring for 4 h, the resulting THF solution of 9-octyl-9-BBN (1.5 eq) was added a solution of **10** (6.1 g, 16.4 mmol) in THF (50 mL), a solution of NaOH (1.97 g, 49.2 mmol, 2.0 eq) in H_2O (20 mL), and Pd(dppf)Cl_2 (402 mg, 0.49 mmol, 3 mol%), and was refluxed for 5 h under N_2 atmosphere. After cooling, the mixture was poured into water and extracted with chloroform. The combined organic layer was separated and concentrated under reduce pressure. The resulting crude product was purified by silica gel column chromatography (chloroform:hexane = 1:9) to afford **4a** (4.0 g, 60 %) as yellow oil.
^{1}H NMR (500 MHz, $CDCl_3$): δ (ppm) 8.38 (s, 1H), 8.31 (s, 1H), 7.92 (d, J = 8.5 Hz, 1H), 7.92 (d, J = 8.5 Hz, 1H), 7.72 (s, 1H), 7.49 (dd, J = 8.5 Hz, 8.5 Hz, 1H), 7.44 (dd, J = 8.5 Hz, 8.5 Hz, 1H), 7.10 (m, 2H), 7.00 (d, J = 8.0 Hz, 1H), 6.95 (d, J = 8.0 Hz, 1H), 2.49 (t, J = 7.5 Hz, 2H), 1.50 (m, 2H), 1.27-1.20 (br, 10H), 0.87 (t, J = 7.5 Hz, 3H). MS (EI mode) m/z 404.

2-Iodo-3-[(3-*n*-octylphenyl)thio]naphtho[2,3-*b*]thiophene (11). To a solution of **4a** (2.0 g, 5.0 mmol) in THF (50 mL) cooled with ice bath was added dropwise n-BuLi (1.6 M hexane solution, 4.7 mL, 7.5 mmol, 1.5 eq) under N_2 atmosphere. After the mixture was stirred for 1 h keeping with ice-bath condition, a

solution of iodine (1.9 g, 7.5 mmol, 1.5 eq) in THF (10 mL) was added slowly to the solution. The resulting mixture was warmed to room temperature and then stirred for 1 h. The mixture was poured into 5% aqueous solution of K_2CO_2 and extracted with chloroform. The combined organic layer was separated and concentrated under reduce pressure. The resulting crude product was purified by silica gel column chromatography (hexane) to afford **11** (1.4 g, 54 %) as pale yellow oil.

^{1}H NMR (500 MHz, $CDCl_3$): δ (ppm) 8.30 (s, 1H), 8.26 (s, 1H), 7.91 (d, J = 8.0 Hz, 1H), 7.88 (d, J = 8.0 Hz, 1H), 7.50 (dd, J = 8.0 Hz, 8.0 Hz, 1H), 7.45 (dd, J = 8.0 Hz, 8.0 Hz, 1H), 7.08 (dd, J = 8.0 Hz, 8.0 Hz, 1H), 7.03 (s, 1H), 6.93 (d, J = 8.0 Hz, 1H), 6.90 (d, J = 8.0 Hz, 1H), 2.49 (t, J = 7.5 Hz, 2H), 1.50 (m, 2H), 1.27-1.20 (br, 10H), 0.87 (t, J = 7.5 Hz, 3H). MS (EI mode) m/z 530.

2-n-Octyl-[1]benzothieno[3,2-*b*]naphtho[2,3-*b*]thiophene (2-C8-BTNT(1a)). A mixture of **11** (1.3 g, 2.45 mmol), $PdCl_2(PPh_3)_2$ (172 mg, 0.25 mmol, 10 mol%), and sodium pivalate hydrate (912 mg, 7.35 mmol, 3.0 eq) in *N*,*N*-dimethylacetamide (50mL) was stirred at 120 °C for 12 h under N_2 atmosphere. After cooling, the reaction mixture was poured into water, and filtered. The brown precipitate was washed with methanol and passed through a silica gel pad with a mixed solution of hexane and chloroform as eluent. The resulting crude product was treated with activated carbon (c.a. 100 mg) in THF solution, and purified by silica gel column chromatography (hexane:chloroform = 1:1). Purification of the product was finally carried out by recrystallization from a mixed solution of chloroform and ethanol at twice times to afford **2-C8-BTNT (1a)** (320 mg, 32 %) as a pale yellow crystal.

^{1}H NMR (500 MHz, $CDCl_3$): δ (ppm) 8.38 (s, 1H), 8.33 (s, 1H), 8.01 (m, 1H), 7.93 (m, 1H), 7.79 (d, J = 8.5 Hz, 1H), 7.75 (s, 1H), 7.51 (m, 2H), 7.31 (d, J = 8.5 Hz,1H), 2.78 (t, J = 7.5 Hz, 2H), 1.71 (tt, J = 7.5 Hz, 2H), 1.38-1.28 (m, 10H), 0.88 (t, J = 7.5 Hz, 3H). MS (EI mode) m/z 402.

Synthesis of 3-octyl-BTNT (1b)

As shown in Scheme S2, **3-C8-BTNT (1b)** was synthesized the same synthetic route as **2-C8-BTNT (1a)** using **3** and 4-bromobenzenthiol as starting materials.

3-[(4-Bromophenyl)thio]naphtho[2,3-*b*]thiophene (12). The title compound was synthesized by a similar procedure of **10** and additional recrystallization from a mixed solution of chloroform and methanol for the reaction of **3** and 4-bromothiophene to afford a yellow crystal (67 %).

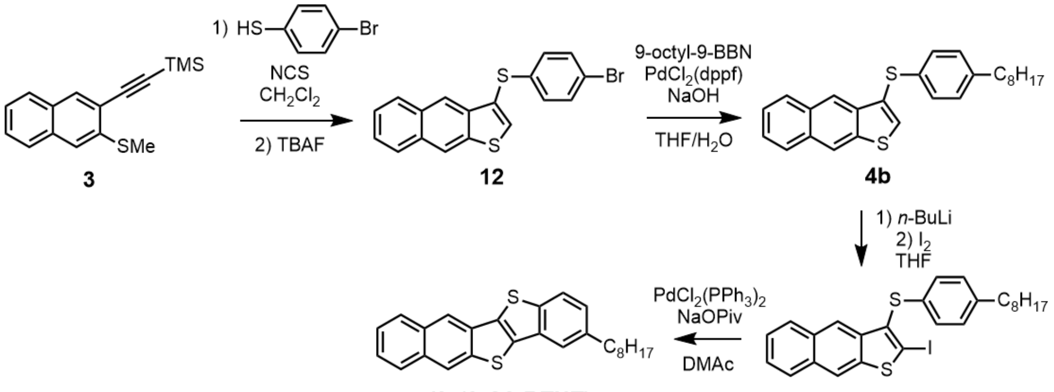

Scheme S2. Synthetic route of **3-C8-BTNT (1b).**

^{1}H NMR (500 MHz, $CDCl_3$): δ (ppm) 8.40 (s, 1H), 8.26 (s, 1H), 7.93 (d, J = 8.5 Hz, 1H), 7.93 (d, J = 8.5 Hz, 1H), 7.83 (s, 1H), 7.50 (dd, J = 8.5 Hz, 8.5 Hz, 1H), 7.47 (dd, J = 8.5 Hz, 8.5 Hz, 1H), 7.31 (d, J = 8.0 Hz, 2H), 7.05 (d, J = 8.0 Hz, 2H). MS (EI mode) m/z 370.

3-[(4-*n*-Octylphenyl)thio]naphtho[2,3-*b*]thiophene (4b). The title compound was synthesized by a similar procedure of **4a** using **12** as a substrate to afford yellow oil (68 %).
^{1}H NMR (500 MHz, $CDCl_3$): δ (ppm) 8.36 (s, 1H), 8.32 (s, 1H), 7.93 (d, J = 8.5 Hz, 1H), 7.91 (d, J = 8.5 Hz, 1H), 7.67 (s, 1H), 7.48 (dd, J = 8.5 Hz, 8.5 Hz, 1H), 7.44 (dd, J = 8.5 Hz, 8.5 Hz, 1H), 7.18 (d, J = 8.0 Hz, 2H), 7.03 (d, J = 8.0 Hz, 2H), 2.51 (t, J = 7.5 Hz, 2H), 1.51 (m, 2H), 1.27-1.20 (br, 10H), 0.86 (t, J = 7.5 Hz, 3H). MS (EI mode) m/z 404.

2-Iodo-3-[(4-*n*-octylphenyl)thio]naphtho[2,3-*b*]thiophene (13). The title compound was synthesized by a similar procedure of **11** using **4b** as a substrate to afford yellow oil (54 %).
^{1}H NMR (500 MHz, $CDCl_3$): δ (ppm) 8.30 (s, 1H), 8.25 (s, 1H), 7.91 (d, J = 8.5 Hz, 1H), 7.88 (d, J = 8.5 Hz, 1H), 7.49 (dd, J = 8.5 Hz, 8.5 Hz, 1H), 7.44 (dd, J = 8.5 Hz, 8.5 Hz, 1H), 7.08 (d, J = 8.0 Hz, 2H), 7.00 (d, J = 8.0 Hz, 2H), 2.49 (t, J = 7.5 Hz, 2H), 1.50 (m, 2H), 1.27-1.20 (br, 10H), 0.87 (t, J = 7.5 Hz, 3H). MS (EI mode) m/z 530.

3-n-Octyl-[1]benzothieno[3,2-*b*]naphtho[2,3-*b*]thiophene (3-C8-BTNT (1b)). The title compound was synthesized by a similar procedure of **2-C8-BTNT (1a)** using **13** as a substrate to afford a pale yellow crystal (27 %).

^{1}H NMR (500 MHz, $CDCl_3$): δ (ppm) 8.39 (s, 1H), 8.34 (s, 1H), 8.01 (m, 1H), 7.93 (m, 1H), 7.84 (d, J = 8.0 Hz, 1H), 7.69 (s, 1H), 7.51 (m, 2H), 7.27 (d, J = 8.0 Hz,1H), 2.79 (t, J = 7.5 Hz, 2H), 1.72 (tt, J = 7.5 Hz, 2H), 1.38-1.28 (m, 10H), 0.89 (t, J = 7.5 Hz, 3H). MS (EI mode) m/z 402.

Synthesis of 8-C8-BTNT (2a)

Scheme S3 shows synthetic route of **8-C8-BTNT (2a)**, which was carried out by the combination of reported procedure [S1-S6] with several modifications and the procedure of **2-C8-BTNT (1a)** (or **3-C8-BTNT (1b)**) as above.

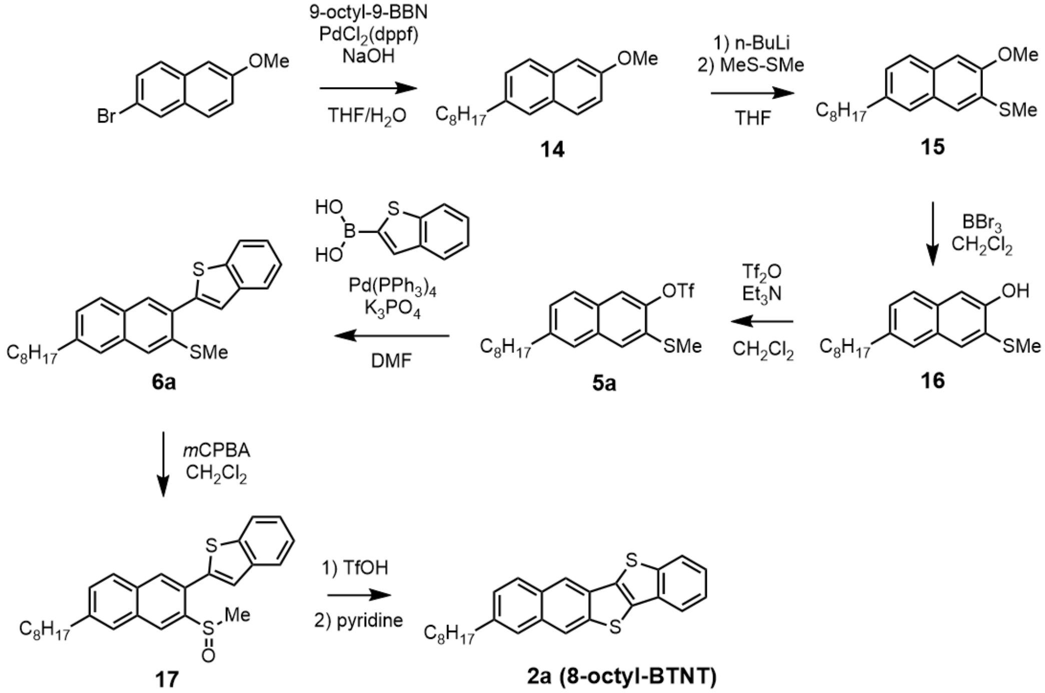

Scheme S3. Synthetic route of **8-C8-BTNT (2a)**.

2-methoxy-6-*n*-octylnaphthalene (14). The title compound was synthesized by a similar procedure of **4a** using 2-methoxy-6-bromonaphthalene as a substrate to afford colorless oil (62 %).

^{1}H NMR (500 MHz, $CDCl_3$): δ (ppm) 7.67 (m, 2H), 7.53 (s, 1H), 7.29 (d, J = 8.0 Hz, 1H), 7.12 (m, 2H), 3.91 (s, 3H), 2.73 (t, J = 7.5 Hz, 2H), 1.68 (tt, J = 7.5 Hz, 2H), 1.36-1.26 (m, 10H), 0.88 (t, J = 7.5 Hz, 3H). MS (EI mode) m/z 270.

2-methoxy-3-methylthio-6-*n*-octylnaphthalene (15). The title compound was synthesized by a similar procedure of **7** using **14** as a substrate to provide a white solid (90 %).

^{1}H NMR (500 MHz, $CDCl_3$): δ (ppm) 7.62 (d, *J* = 8.0 Hz, 1H), 7.49 (s, 1H), 7.41 (s, 1H), 7.23 (d, *J* = 8.0 Hz, 2H), 7.05 (s, 1H), 3.98 (s, 3H), 2.72 (t, *J* = 7.5 Hz, 2H), 2.53 (s, 3H), 1.68 (tt, *J* = 7.5 Hz, 2H), 1.35-1.26 (m, 10H), 0.88 (t, *J* = 7.5 Hz, 3H). MS (EI mode) m/z 316.

2-hydroxy-3-methylthio-6-*n*-octylnaphthalene (16). The title compound was synthesized by a similar procedure of **8** using **15** as a substrate to provide a white solid (92 %).
^{1}H NMR (500 MHz, $CDCl_3$): δ (ppm) 7.93 (s, 1H), 7.61 (d, *J* = 8.5 Hz, 1H), 7.48 (s, 1H), 7.28 (m, 2H), 6.56 (s, 1H), 2.71 (t, *J* = 7.5 Hz, 2H), 2.42 (s, 3H), 1.67 (tt, *J* = 7.5 Hz, 2H), 1.37-1.26 (m, 10H), 0.88 (t, *J* = 7.5 Hz, 3H). MS (EI mode) m/z 302.

3-Methylthio-6-*n*-octylnaphthalen-2-yl trifluoromethanesulfonate (5a). The title compound was synthesized by a similar procedure of **9** using **16** as a substrate to provide a white solid (74 %).
^{1}H NMR (500 MHz, $CDCl_3$): δ (ppm) 7.73 (d, *J* = 8.5 Hz, 1H), 7.68 (s, 1H), 7.64 (s, 1H), 7.56 (s, 1H), 7.36 (d, *J* = 8.5 Hz, 1H), 2.76 (t, *J* = 7.5 Hz, 2H), 2.59 (s, 3H), 1.69 (tt, *J* = 7.5 Hz, 2H), 1.35-1.26 (m, 10H), 0.88 (t, *J* = 7.5 Hz, 3H). MS (EI mode) m/z 434.

2-(3-Methylthio-6-*n*-octylnaphthalen-2-yl)benzo[*b*]thiophene (6a). A mixture of **5a** (1.5 g, 3.45 mmol), benzo[*b*]thiophene-2-boronic acid (922mg, 5.18 mmol, 1.5 eq), tripotassium phosphate (1.83 g, 8.63 mmol, 2.5 eq), and $Pd(PPh_3)_4$ (199 mg, 0.17 mmol, 5 mol%) in *N*,*N*-dimethylformamide (50mL) was stirred at 80 °C for 12 h under N_2 atmosphere. After cooling, the reaction mixture was poured into water and extracted with chloroform. The combined organic layer was separated and concentrated under reduce pressure. The resulting crude product was passed through a silica gel pad with a mixed solution of hexane and chloroform as eluent. The resulting crude product was purified by silica gel column chromatography (hexane:chloroform = 4:1) to afford **6a** (1.22 g, 84 %) as a pale yellow solid.
^{1}H NMR (500 MHz, $CDCl_3$): δ (ppm) 7.88 (s, 1H), 7.86 (d, *J* = 8.0 Hz, 1H), 7.82 (d, *J* = 8.0 Hz, 1H), 7.72 (d, *J* = 8.5 Hz, 1H), 7.57 (s, 1H), 7.56 (s, 1H), 7.54 (s, 1H), 7.38 (dd, *J* = 8.0 Hz, , 1H), 7.38 (d, *J* = 8.0 Hz, 8.0 Hz, 1H), 7.35 (d, *J* = 8.0 Hz, 8.0 Hz, 1H), 2.77 (t, *J* = 7.5 Hz, 2H), 2.52 (s, 3H), 1.71 (tt, *J* = 7.5 Hz, 2H), 1.35-1.25 (m, 10H), 0.88 (t, *J* = 7.5 Hz, 3H). MS (EI mode) m/z 418.

8-n-Octyl-[1]benzothieno[3,2-*b*]naphtho[2,3-*b*]thiophene (8-C8-BTNT (2a)). To a solution of **6a** (1.2 g, 2.76 mmol) in dichloromethane (50 mL) cooled with ice bath was added *m*-chloroperoxy benzoic acid (containing c.a. 30 % water, 717 mg, 1.0 eq). After the mixture was warmed to room temperature and then stirred for 3 h. The reaction mixture was poured into 5 % aqueous solution of $NaHCO_3$ and stirred for 1h.

The brown precipitate was filtered and washed with water to afford the crude product of **17**, which used without further purification. A mixture of the crude product of **17** (1.2 g) and trifluoromethanesulfonic acid (5 g) was stirred for 12 h at room temperature. The reaction mixture was poured into ice-water. The brown precipitate was filtered and washed with water. The residue was dissolved in pyridine (30 mL), and the resulting mixture was refluxed for 3 h. After cooling, the reaction mixture was poured into methanol, and the yellow precipitate was filtered and washed with methanol. The resulting crude product was treated with activated carbon (c.a. 100 mg) in THF solution at room temperature, and purified by silica gel column chromatography (hexane:chloroform = 1:1). Purification of the product was finally carried out by recrystallization from a mixed solution of chloroform and ethanol at twice times to afford **8-C8-BTNT (2a)** (430 mg, 39 % (2 steps)) as a pale yellow crystal.
^{1}H NMR (500 MHz, $CDCl_3$): δ (ppm) 8.31 (s, 1H), 8.31 (s, 1H), 7.95 (d, J = 8.5 Hz, 1H), 7.93 (d, J = 8.5 Hz, 1H), 7.88 (d, J = 8.5 Hz, 1H), 7.68 (s, 1H), 7.48 (dd, J = 8.5 Hz, 8.5 Hz, 1H), 7.42 (dd, J = 8.5 Hz, 8.5 Hz, 1H), 7.38 (d, J = 8.5 Hz,1H), 2.81 (t, J = 7.5 Hz, 2H), 1.74 (tt, J = 7.5 Hz, 2H), 1.40-1.28 (m, 10H), 0.88 (t, J = 7.5 Hz, 3H). MS (EI mode) m/z 402.

Synthesis of 9-C8-BTNT (2b)

As shown in Scheme S4, **9-C8-BTNT (2b)** was synthesized the same synthetic route as **8-C8-BTNT (2a)** using **17** as starting materials.

7-methoxynaphthalene-2-yl trifluoromethanesulfonate (18). The title compound was synthesized by a similar procedure of **9** using 7-methoxy-2-naphthol as a substrate to provide colorless oil (96 %).
^{1}H NMR (500 MHz, $CDCl_3$): δ (ppm) 7.82 (d, J = 9.0 Hz, 1H), 7.77 (d, J = 9.0 Hz, 1H), 7.64 (d, J = 2.5 Hz, 1H), 7.22 (dd, J = 9.0 Hz, 2.5 Hz, 1H), 7.21 (dd, J = 9.0 Hz, 2.5 Hz, 1H), 7.14 (d, J = 2.5 Hz, 1H), 3.93 (s, 3H). MS (EI mode) m/z 306.

2-methoxy-7-*n*-octylnaphthalene (19). The title compound was synthesized by a similar procedure of **4a** using **18** as a substrate to provide colorless oil (92 %).
^{1}H NMR (500 MHz, $CDCl_3$): δ (ppm) 7.69 (m, 2H), 7.51 (s, 1H), 7.18 (dd, J = 8.5 Hz, 1.5 Hz, 1H), 7.08 (s, 1H), 7.06 (m, 1H), 3.93 (s, 3H), 2.74 (t, J = 7.5 Hz, 2H), 1.67 (tt, J = 7.5 Hz, 2H), 1.36-1.26 (m, 10H), 0.88 (t, J = 7.5 Hz, 3H) . MS (EI mode) m/z 270.

2-methoxy-3-methylthio-7-*n*-octylnaphthalene (20). The title compound was synthesized by a similar procedure of **7** using **19** as a substrate to afford colorless oil (70 %).

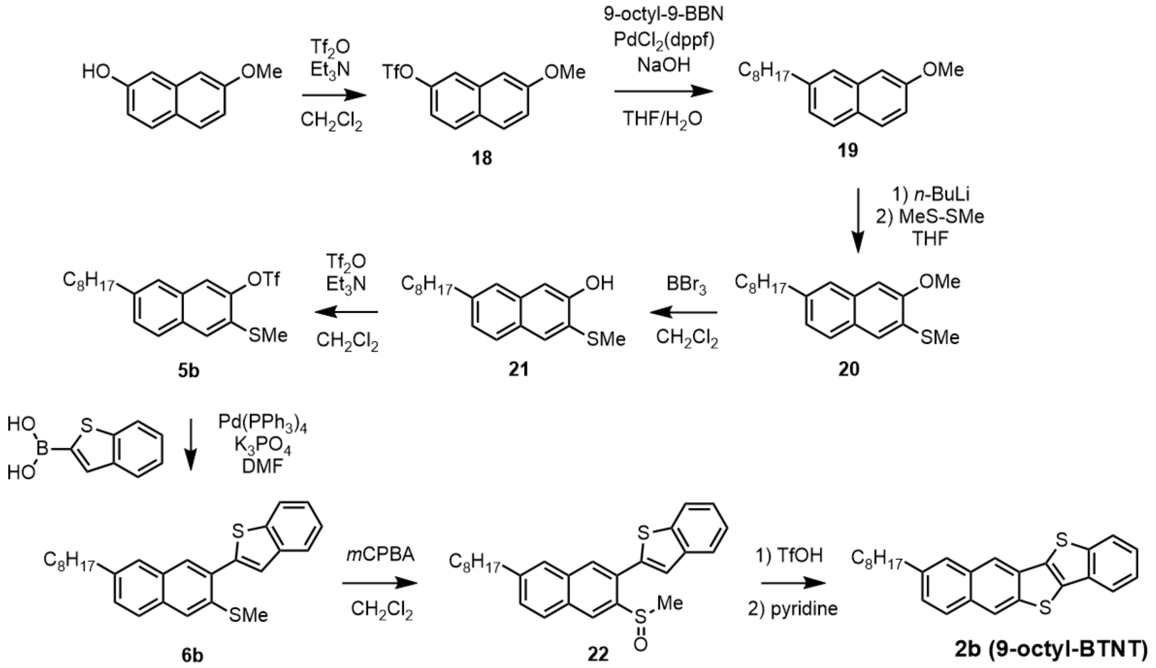

Scheme S4. Synthetic route of **9-C8-BTNT.**

^{1}H NMR (500 MHz, $CDCl_3$): δ (ppm) 7.61 (d, J = 8.0 Hz, 1H), 7.47 (s, 1H), 7.44 (s, 1H), 7.18 (d, J = 8.0 Hz, 1H), 7.03 (s, 1H), 3.99 (s, 3H), 2.72 (t, J = 7.5 Hz, 2H), 2.52 (s, 3H), 1.67 (tt, J = 7.5 Hz, 2H), 1.35-1.26 (m, 10H), 0.88 (t, J = 7.5 Hz, 3H). MS (EI mode) m/z 316.

2-hydroxy-3-methylthio-6-*n*-octylnaphthalene (21). The title compound was synthesized by a similar procedure of **8** using **20** as a substrate to provide a white solid (88 %).
^{1}H NMR (500 MHz, $CDCl_3$): δ (ppm) 7.97 (s, 1H), 7.63 (d, J = 8.0 Hz, 1H), 7.45 (s, 1H), 7.25 (s, 1H), 7.17 (d, J = 8.0 Hz, 1H), 6.63 (s, 1H), 2.72 (t, J = 7.5 Hz, 2H), 2.40 (s, 3H), 1.68 (tt, J = 7.5 Hz, 2H), 1.37-1.26 (m, 10H), 0.87 (t, J = 7.5 Hz, 3H). MS (EI mode) m/z 302.

3-Methylthio-7-*n*-octylnaphthalen-2-yl trifluoromethanesulfonate (5b). The title compound was synthesized by a similar procedure of **9** using **21** as a substrate to provide a white solid (85 %).
^{1}H NMR (500 MHz, $CDCl_3$): δ (ppm) 7.71 (d, J = 8.5 Hz, 1H), 7.68 (s, 1H), 7.66 (s, 1H), 7.57 (s, 1H), 7.39 (d, J = 8.5 Hz, 1H), 2.75 (t, J = 7.5 Hz, 2H), 2.58 (s, 3H), 1.69 (tt, J = 7.5 Hz, 2H), 1.35-1.26 (m, 10H), 0.88 (t, J = 7.5 Hz, 3H). MS (EI mode) m/z 434.

2-(3-Methylthio-7-*n*-octylnaphthalen-2-yl)benzo[*b*]thiophene (6b). The title compound was synthesized by a similar procedure of **6a** using **5b** as a substrate to provide pale yellow oil (55 %).
^{1}H NMR (500 MHz, $CDCl_3$): δ (ppm) 7.87 (m, 2H), 7.82 (d, *J* = 8.0 Hz, 1H), 7.71 (d, *J* = 8.0 Hz, 1H), 7.62 (s, 1H), 7.57 (s, 1H), 7.54 (s, 1H), 7.36 (m, 3H), 2.75 (t, *J* = 7.5 Hz, 2H), 2.51 (s, 3H), 1.69 (tt, *J* = 7.5 Hz, 2H), 1.35-1.25 (m, 10H), 0.88 (t, *J* = 7.5 Hz, 3H). MS (EI mode) m/z 418.

9-n-Octyl-[1]benzothieno[3,2-*b*]naphtho[2,3-*b*]thiophene (9-C8-BTNT (2b)). The title compound was synthesized by a similar procedure of **8-C8-BTNT (2a)** using **6b** as a substrate to provide a pale yellow crystal (32 %).
^{1}H NMR (500 MHz, $CDCl_3$): δ (ppm) 8.34 (s, 1H), 8.29 (s, 1H), 7.95 (d, *J* = 8.0 Hz, 1H), 7.89 (d, *J* = 8.0 Hz, 1H), 7.85 (d, *J* = 8.0 Hz, 1H), 7.78 (s, 1H), 7.48 (dd, *J* = 8.0 Hz, 8.0 Hz, 1H), 7.43 (dd, *J* = 8.0 Hz, 8.0 Hz, 1H), 7.38 (d, *J* = 8.0 Hz,1H), 2.82 (t, *J* = 7.5 Hz, 2H), 1.74 (tt, *J* = 7.5 Hz, 2H), 1.39-1.28 (m, 10H), 0.88 (t, *J* = 7.5 Hz, 3H). MS (EI mode) m/z 402.

3. Structural Characteristics

3.1 Crystallographic parameters.

Table S1. Crystallographic parameters of *mono*-C8-BTNT regioisomers.

	**1a**	**1b**	**2a**	**2b**
Chemical formula	$C_{26}H_{26}S_2$	$C_{26}H_{26}S_2$	$C_{26}H_{26}S_2$	$C_{26}H_{26}S_2$
Formula weight	402.61	402.61	402.61	402.61
Crystal system	triclinic	triclinic	triclinic	triclinic
Space group	$P\bar{1}$ (#2)	$P\bar{1}$ (#2)	$P\bar{1}$ (#2)	$P\bar{1}$ (#2)
a (Å)	6.01908 (11)	6.0576 (3)	6.00978 (14)	6.1144 (3)
b (Å)	7.87883 (16)	8.0009 (4)	7.87052 (18)	7.9480 (5)
c (Å)	45.2168 (12)	44.519 (3)	45.2747 (12)	44.660 (2)
α (deg)	91.9646 (19)	87.629 (5)	91.737 (2)	87.923 (5)
β (deg)	92.9466 (18)	88.944 (4)	92.907 (2)	89.367 (4)
γ (deg)	90.3409 (16)	89.750 (4)	90.2170 (18)	89.762 (5)
V (Å^3)	2140.17 (8)	2155.4 (2)	2137.73 (9)	2168.8 (2)
Z value	4	4	4	4
D_{calc} (g/cm^3)	1.249	1.241	1.251	1.233
Temperature (K)	300	300	300	300
Radiation	CuKα	MoKα	MoKα	CuKα
No. of reflections	7256	9101	9624	7410
No. of variables	505	505	505	505
Residuals, R (I>2s(I))	0.0638	0.1215	0.0648	0.0763
Residuals, wR^2 (all reflections)	0.1766	0.3749	0.1583	0.2374
Goodness of fit indicator	1.092	1.048	0.980	0.951

3.2. Intralayer molecular packing.

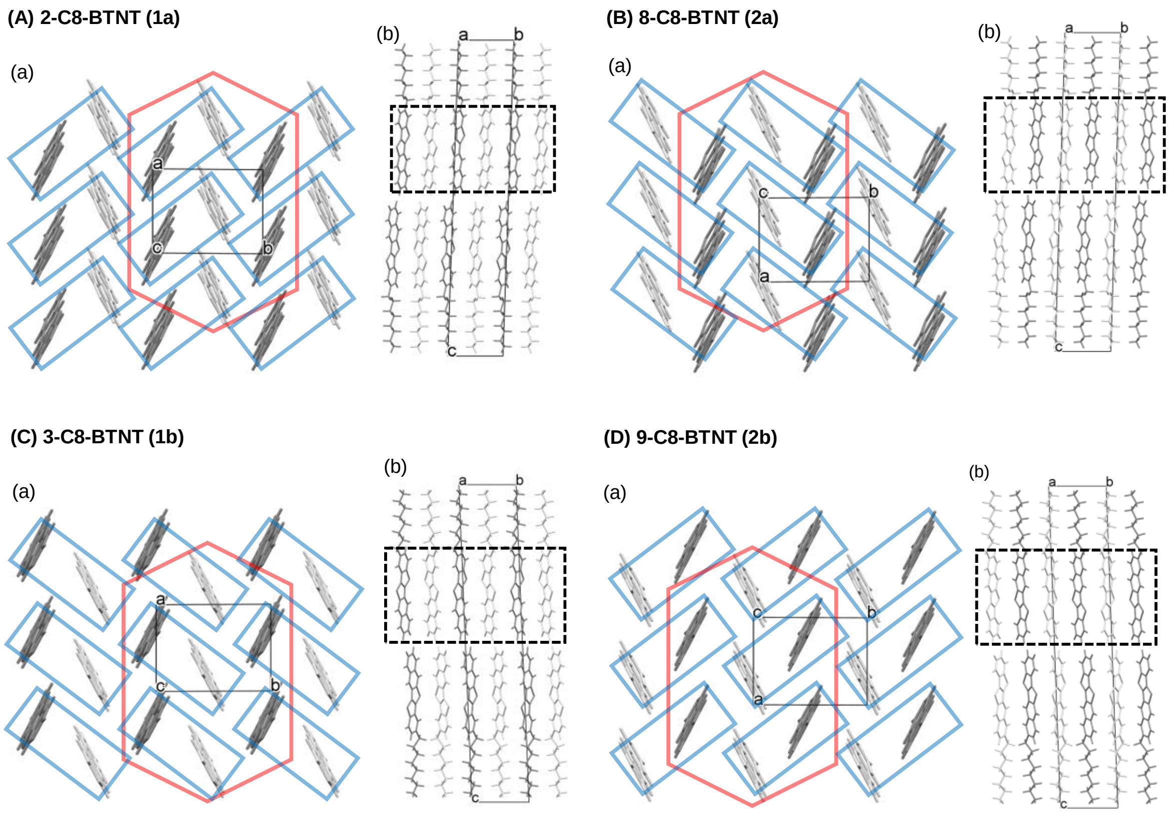

Figure S1. Intralayer molecular packing motifs of *mono*-C8-BTNT isomers of (a) **1a**, (b) **1b**, (c) **2a**, (d) **2b** over an extended area from Figures 2 and 3. Each left figure shows the arrangement of BTNT cores projected to *a*-*b* plane, where a red hexagon encloses 6 nearest neighboring molecules of the central molecule, and blue rectangles enclose crystallographically independent molecular pairs. Each right figure shows the molecular arrangement projected to *b*-*c* plane.

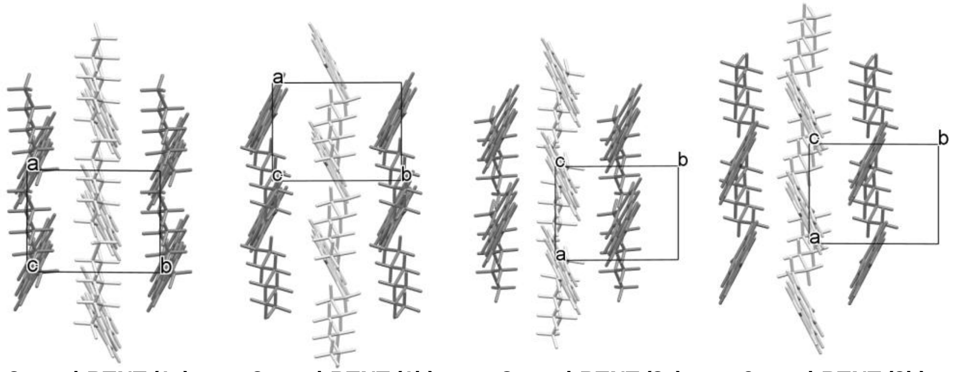

2-octyl-BTNT (1a) **3-octyl-BTNT (1b)** **8-octyl-BTNT (2a)** **9-octyl-BTNT (2b)**

Figure S2. Intralayer molecular packings of all the regioisomers of *mono*-C8-BTNTs of (a) **1a**, (b) **1b**, (c) **2a**, (d) **2b**, projected to the *a*-*b* plane.

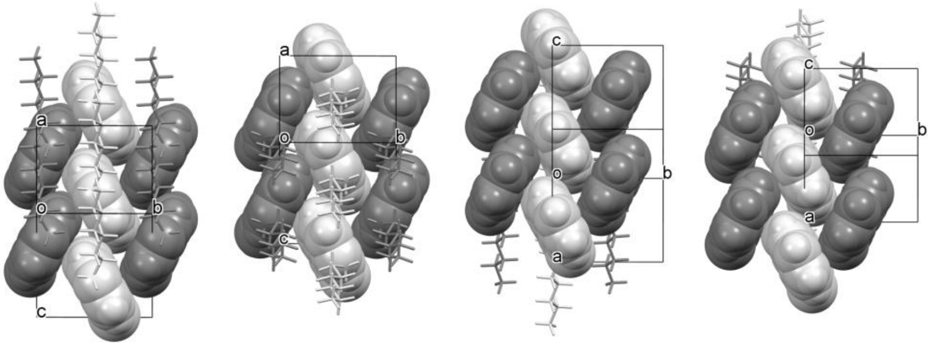

1a **1b** **2a** **2b**

Figure S3. Packing diagrams of BTNT cores projected along the long axis of cores for all the regioisomers of *mono*-C8-BTNTs of (a) **1a**, (b) **1b**, (c) **2a**, (d) **2b**.

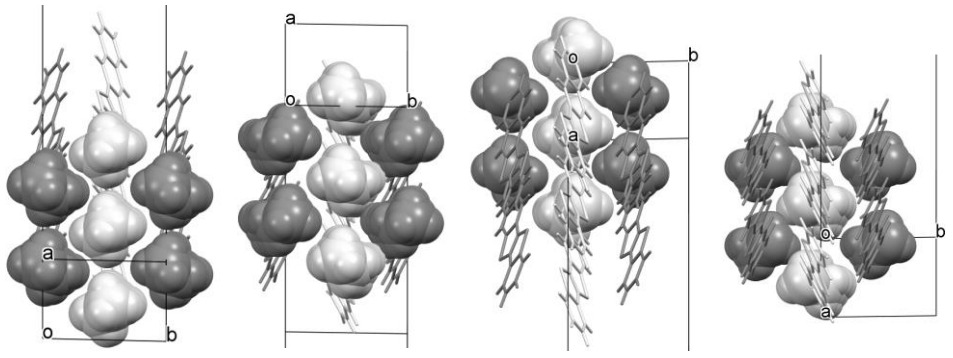

1a **1b** **2a** **2b**

Figure S4. Packing diagrams of substituted octyl chains projected along the long axis of chains for all the regioisomers of *mono*-C8-BTNTs of (a) **1a**, (b) **1b**, (c) **2a**, (d) **2b**.

3.2. Atomic distances.

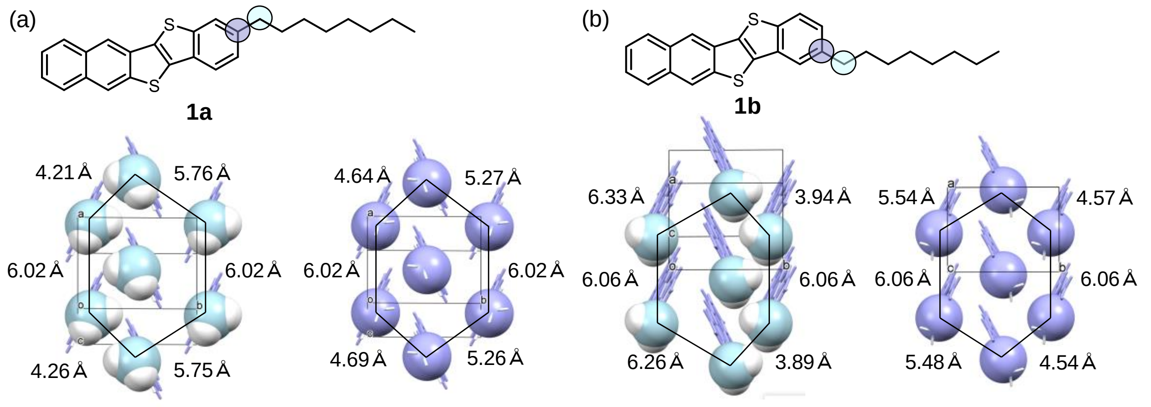

Figure S5. Intralayer arrangement of specific carbon atoms around the substituting positions for *mono*-C8-BTNTs of (a) **1a**, and (b) **1b**. Each left figure shows the arrangement of intrachain carbon closest to the substituting position shown by space-filled light blue color. Each right figure shows the arrangement of sp^2 carbon within the BTNT core at the substituting position shown by space-filled purple color. The positional arrangements of these atoms are more uneven for the intrachain carbon and **1b**.

3. Intermolecular Transfer Integrals.

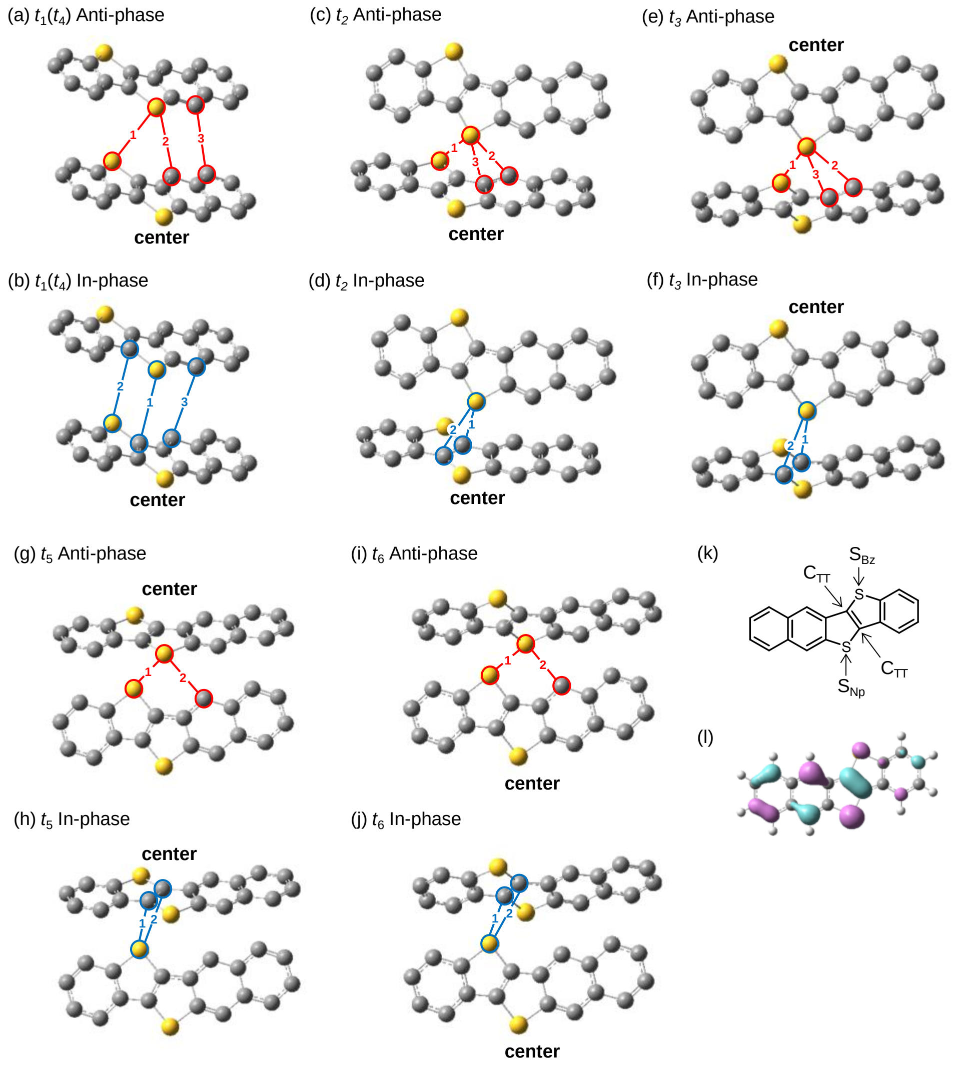

Figure S6. (a)-(j) Atomic pairs that have the largest two or three contributions to the intermolecular transfer integrals. (k) Each intramolecular atoms were specified by C_{TT} (TT: thienothophene), S_{Bz} (Bz: close to benzene), and S_{Np} (Np: close to naphthalene). These notations are used in Table S3–S6. (l) Highest occupied molecular orbital (HOMO) of the BTNT core obtained by DFT calculation (B3LYP/6-31G* level.).

Table S2. Contributions of each atom pairs to the intermolecular transfer integrals in the crystals of **1a**. See Figure S7 for notations of C_{TT}, S_{Bz}, and S_{Np}.

Pair	Molecular pair transfer integrals (meV)		Atomic pairs of transfer integrals having the large contribution				
	Molecular pair[a)]	anti-phase (+) or in-phase (-)	Figure	Atomic pair	Contact type / distance (Å)	Values (meV)	Ratio (%) Within the phase
t_1	64.0	151.0	(a)	S_{Np}-S_{Bz}	1 / 3.60	50.8	34
				S_{Np}-C	2 / 3.65	42.6	28
		-87.1	(b)	S_{Np}-C_{TT}	1 / 3.96	-26.2	30
				S_{Bz}-C_{TT}	2 / 4.00	-16.1	18
				C-C	3 / 3.91	-11.2	13
t_2	-50.4	204.6	(c)	S_{Np}-C	2 / 4.11	36.3	18
				S_{Np}-S_{Bz}	1 / 4.30	32.0	16
				S_{Np}-C	3 / 3.45	26.1	13
		-255.0	(d)	S_{Np}-C_{TT}	1 / 3.46	-93.4	37
				S_{Np}-C_{TT}	2 / 3.54	-52.8	21
t_3	-39.5	202.2	(e)	S_{Np}-C	2 / 4.11	36.6	18
				S_{Np}-C	3 / 3.43	35.5	18
				S_{Np}-S_{Bz}	1 / 4.29	24.4	12
		-241.7	(f)	S_{Np}-C_{TT}	1 / 3.44	-100.3	19
				S_{Np}-C_{TT}	2 / 3.52	-45.3	41
t_5	-41.9	156.6	(g)	S_{Np}-S_{Bz}	1 / 4.29	26.7	17
		-198.6	(h)	S_{Bz}-C_{TT}	1 / 3.48	-45.7	23
				S_{Bz}-C_{TT}	2 / 3.52	-31.9	16
t_6	-19.9	165.5	(i)	S_{Np}--S_{Bz}	1 / 4.40	27.1	16
				S_{Np}-C	2 / 4.51	18.3	11
		-185.4	(j)	S_{Bz}-C_{TT}	1 / 3.55	-48.7	26
				S_{Bz}-C_{TT}	2 / 3.65	-29.0	16

Table S3. Contributions of each atom pairs to the intermolecular transfer integrals in the crystals of **2a**. See Figure S7 for notations of C_{TT}, S_{Bz}, and S_{Np}.

Pair	Molecular pair transfer integrals (meV)		Atomic pairs transfer integral having the large contribution				
	Molecular pair[a)]	anti-phase (+) or in-phase (-)	Figure	Atomic pair	Contact type / Distance (Å)	Values (meV)	Ratio (%) Within the phase
***t*₁**	59.9	141.9	(a)	S_{Np}-S_{Bz}	1 / 3.56	43.6	31
				S_{Np}-C	2 / 3.67	40.6	29
				C-C	3 / 3.62	15.6	11
		-82.0	(b)	S_{Np}-C_{TT}	1 / 3.96	-24.9	30
				S_{Bz}-C_{TT}	2 / 3.95	-13.0	16
				C-C	3 / 3.95	-11.4	14
***t*₂**	-53.2	208.4	(c)	S_{Np}-C	3 / 3.49	33.4	16
				S_{Np}-C	2 / 4.15	32.0	15
				S_{Np}-S_{Bz}	1 / 4.25	30.4	15
		-261.6	(d)	S_{Np}-C_{TT}	1 / 3.44	-96.5	37
				S_{Np}-C_{TT}	2 / 3.49	-51.9	20
***t*₃**	-30.9	201.5	(e)	S_{Np}-C	2 / 4.04	38.7	19
				S_{Np}-C	3 / 3.44	33.0	16
				S_{Np}-S_{Bz}	1 / 4.36	24.5	12
		-232.4	(f)	S_{Np}-C_{TT}	1 / 3.49	-86.4	37
				S_{Np}-C_{TT}	2 / 3.61	-39.5	17
***t*₅**	-31.3	151.9	(g)	S_{Np}-S_{Bz}	1 / 4.30	27.1	18
				S_{Np}-C	2 / 4.58	15.0	10
		-183.2	(h)	S_{Bz}-C_{TT}	1 / 3.44	-49.3	27
				S_{Bz}-C_{TT}	2 / 3.54	-28.3	15
***t*₆**	-29.3	155.7	(i)	S_{Np}--S_{Bz}	1 / 4.31	26.6	17
				S_{Np}-C	2 / 4.60	15.0	10
		-185.0	(j)	S_{Bz}-C_{TT}	1 / 3.45	-49.7	27
				S_{Bz}-C_{TT}	2 / 3.54	-28.7	16

Table S4. Contributions of each atom pairs to the intermolecular transfer integrals in the crystals of **1b**. See Figure S7 for notations of C_{TT}, S_{Bz}, and S_{Np}.

Pair	Molecular pair transfer integrals (meV)		Atomic pairs of transfer integrals having the large contribution				
	Molecular pair[a)]	anti-phase (+) or in-phase (-)	Figure	Atom pair	Contact type / distance (Å)	Values (meV)	Ratio (%) Within the phase
t_1	58.3	143.1	(a)	S_{Np}-S_{Bz}	1 / 3.56	53.0	37
				S_{Np}-C	2 / 3.78	33.8	24
		-84.8	(b)	S_{Np}-C_{TT}	1 / 4.00	-26.1	31
				S_{Bz}-C_{TT}	2 / 4.00	-15.5	18
				C-C	3 / 3.98	-10.1	12
t_2	-49.7	184.0	(c)	S_{Np}-C	2 / 4.14	32.1	17
				S_{Np}-S_{Bz}	1 / 4.32	31.2	17
				S_{Np}-C	3 / 3.49	21.8	12
		-233.7	(d)	S_{Np}-C_{TT}	1 / 3.49	-94.1	40
				S_{Np}-C_{TT}	2 / 3.57	-48.5	21
t_3	-51.0	173.3	(e)	S_{Np}-C	2 / 4.13	33.0	19
				S_{Np}-S_{Bz}	1 / 4.33	27.4	16
				S_{Np}-C	3 / 3.49	20.1	12
		-224.3	(f)	S_{Np}-C_{TT}	1 / 3.45	-92.6	41
				S_{Np}-C_{TT}	2 / 3.58	-41.3	18
t_5	-19.3	157.2	(g)	S_{Np}-S_{Bz}	1 / 4.38	26.6	17
		-176.5	(h)	S_{Bz}-C_{TT}	1 / 3.50	-50.2	28
				S_{Bz}-C_{TT}	2 / 3.63	-30.2	17
t_6	-48.3	161.1	(i)	S_{Np}-S_{Bz}	1 / 4.25	36.2	22
		-209.4	(j)	S_{Bz}-C_{TT}	1 / 3.44	-65.5	31
				S_{Bz}-C_{TT}	2 / 3.52	-38.6	18

Table S5. Contributions of each atom pairs to the intermolecular transfer integrals in the crystals of **2b**. See Figure S7 for notations of C_{TT}, S_{Bz}, and S_{Np}.

Pair	Molecular pair transfer integrals (meV)		Atomic pairs of transfer integrals having the large contribution				
	Molecular pair[a)]	anti-phase (+) or anti-phase (-)	Figure	Atom pair	Contact type / distance (Å)	Values (meV)	Ratio (%) Within the phase
t_1	56.2	137.1	(a)	S_{Np}-S_{Bz}	1 / 3.58	46.0	34
				S_{Np}-C	2 / 3.82	34.5	25
				C-C	3.73	13.9	10
		-80.8	(b)	S_{Np}-C_{TT}	1 / 4.04	-22.8	28
				C-C	3 / 4.00	-11.0	14
				S_{Bz}-C_{TT}	2 / 4.08	-10.2	13
t_2	-46.9	191.6	(c)	S_{Np}-C	2 / 4.05	40.4	21
				S_{Np}-C	3 / 3.47	27.5	14
				S_{Np}-S_{Bz}	1 / 4.38	25.1	13
		-238.5	(d)	S_{Np}-C_{TT}	1 / 3.52	-83.9	35
				S_{Np}-C_{TT}	2 / 3.66	-35.4	15
t_3	-75.3	192.5	(e)	S_{Np}-S_{Bz}	1 / 4.25	33.3	17
				S_{Np}-C	2 / 4.21	30.9	16
				S_{Np}-C	3 / 3.54	25.1	13
		-267.8	(f)	S_{Np}-C_{TT}	1 / 3.46	-97.6	36
				S_{Np}-C_{TT}	2 / 3.51	-45.6	17
t_5	-24.6	155.3	(g)	S_{Np}-S_{Bz}	1 / 4.30	29.4	19
		-179.9	(h)	S_{Bz}-C_{TT}	1 / 3.46	-43.0	24
				S_{Bz}-C_{TT}	2 / 3.56	-28.0	16
t_6	-25.5	151.4	(i)	S_{Np}-S_{Bz}	1 / 4.29	28.5	19
		-177.0	(j)	S_{Bz}-C_{TT}	1 / 3.44	-43.4	25
				S_{Bz}-C_{TT}	2 / 3.56	-25.4	14

4. Thermal Characteristics

Phase transition enthalpy (ΔH) was obtained by integrating the area under the DSC curve (Figure 7) around the transition, and phase transition entropy (ΔS) was by $\Delta S = \Delta H/T_{trans}$, where T_{trans} is the transition temperature[S7]. All the values for *mono*-C8-BTNTs regioisomers are summarized in Table S1 and Table S2. In addition, the total enthalpies (ΔH_{total}) estimated from respective peaks of the DSC charts are shown in Figure S1.

Table S6. Thermal parameters of *mono*-C8-BTNTs calculated from DSC charts at 1st scan.

	**Heating process**			**Cooling process**		
	Transition temp. (°C)	ΔH (KJ/mol)	ΔS (J/mol・K)	Transition temp. (°C)	ΔH (KJ/mol)	ΔS (J/mol・K)
**1a**	180.0	19.2	42.3	118.4	18.2	46.5
	221.9	19.7	39.9	218.7	18.9	38.5
**1b**	144.8	13.4	32.0	73.6	3.1	9.0
	168.4	17.5	39.7	164.9	17.6	40.3
**2a**	194.8	17.4	37.3	133.1	17.1	42.1
	221.3	18.6	37.6	218.9	17.9	36.3
**2b**	110.2	0.7	1.8	80.8	8.9	25.3
	160.4	37.2	85.8	156.3	18.3	42.6

Table S7. Thermal parameters of *mono*-C8-BTNTs calculated from DSC charts at 1st scan.

	**Heating process**			**Cooling process**		
	Transition temp. (°C)	ΔH (KJ/mol)	ΔS (J/mol・K)	Transition temp. (°C)	ΔH (KJ/mol)	ΔS (J/mol・K)
**1a**	176.1	18.6	41.4	131.8	19.2	47.3
	221.0	18.7	37.9	219.2	18.8	38.1
**1b**	86.0	4.3	11.9	74.7	2.8	8.0
	138.9	5.8	14.2	165.9	16.8	38.2
	168.1	16.8	38.0			
**2a**	188.1	17.7	38.4	146.7	18.7	44.5
	221.0	17.9	36.2	219.3	17.8	36.1
**2b**	106.0	4.0	10.5	103.4	12.7	33.8
	159.1	36.1	83.5	156.7	18.2	42.2

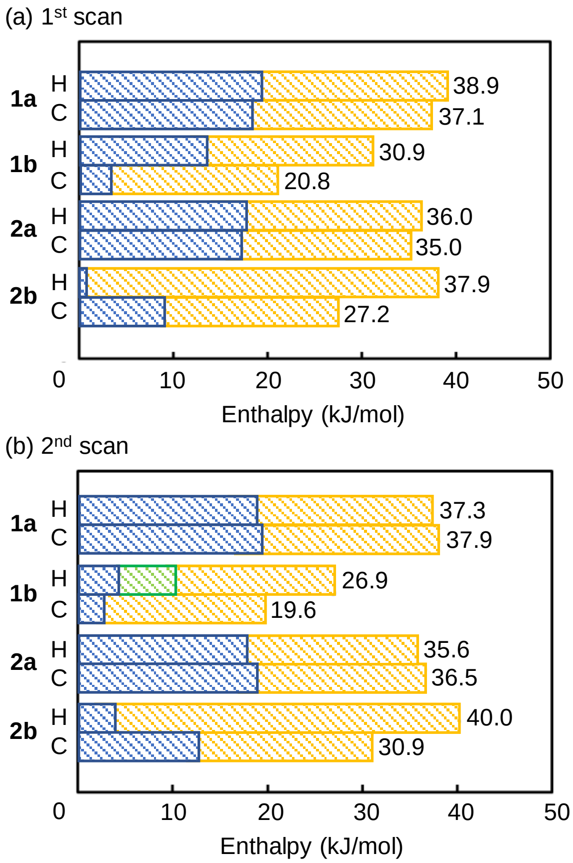

Figure S7. Enthalpy changes of *mono*-C8-BTNT regioisomers estimated at (a) 1st scan and (b) 2nd scan of the DSC curves. Notations are follows; H: calculated from heating process, C: calculated from cooling process, Blue and Green: crystal to liquid-crystal (or crystal to crystal) phase transition, Orange: melting point. The values shown on the right side of each bars are the total enthalpy (ΔH_{total}).

5. Device Characterization.

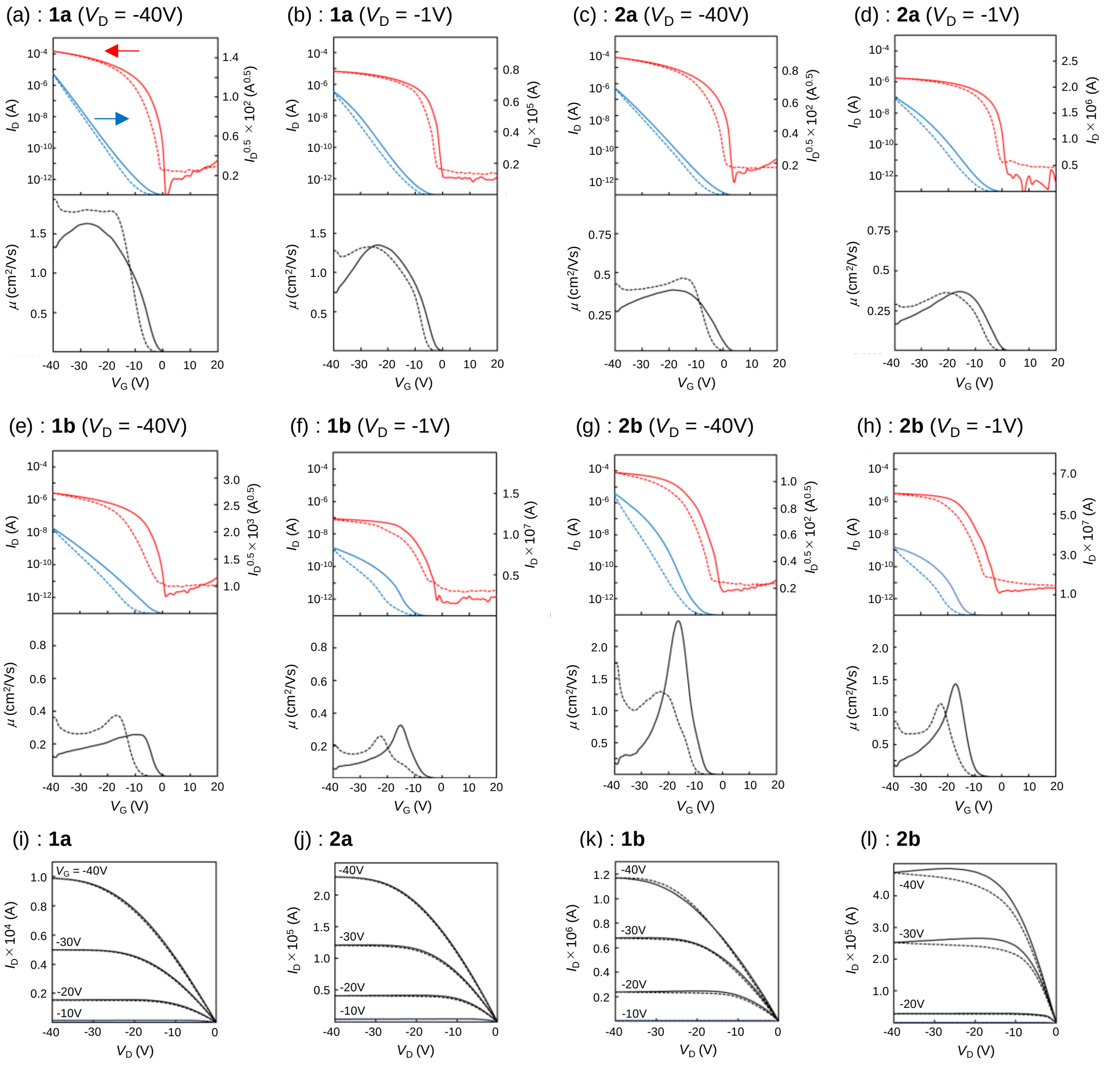

Figure S8. Typical device characteristics of spin-coated polycrystalline TFTs for all the *mono*-C8-BTNT regioisomers of **1a**, **1b**, **2a**, and **2b**. Each film was annealed at 120 ºC for 5 min. (a)(c)(e)(g) transfer characteristics in the saturation regime and plots of their mobilities as a function of V_G, (b)(d)(f)(h) transfer characteristics in the linear regime and plots of their mobilities as a function of V_G, and (i)–(l) output characteristics. Solid lines and dashed lines are forward and backward scans, respectively.

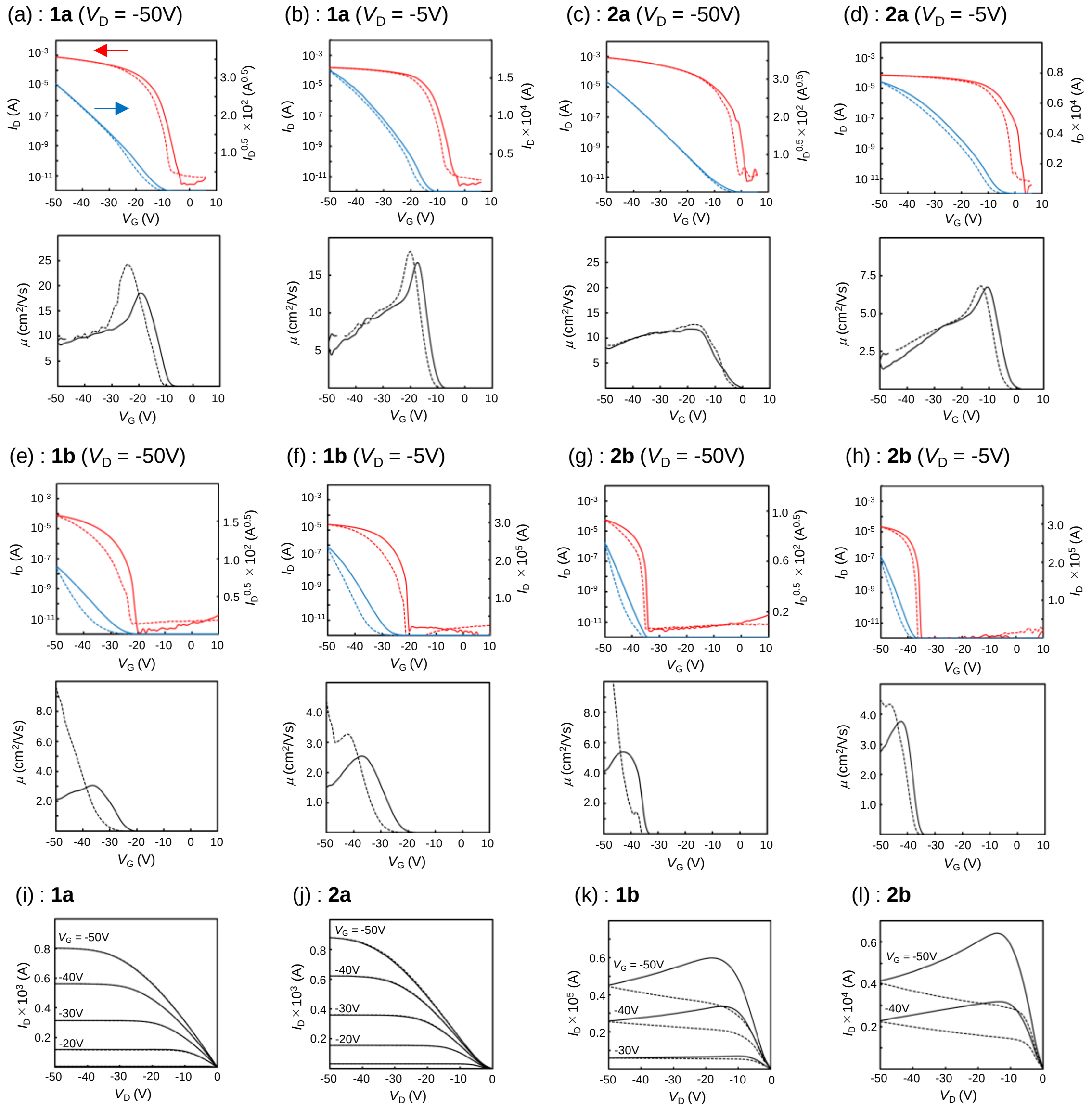

Figure S9. Typical device characteristics of single crystalline TFTs for all the *mono*-C8-BTNT regioisomers of **1a**, **1b**, **2a**, and **2b**. (a)(c)(e)(g) transfer characteristics in the saturation regime and plots of their mobilities as a function of V_G, (b)(d)(f)(h) transfer characteristics in the linear regime and plots of their mobilities as a function of V_G, and (i)–(l) output characteristics. Solid lines and dashed lines are forward and backward scans, respectively.

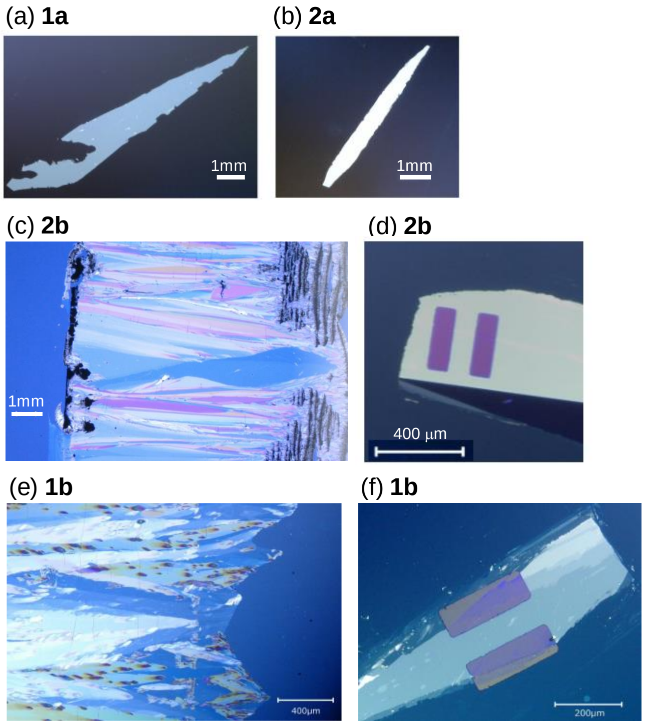

Figure S10. Optical microscope images of single crystalline thin films and devices with *mono*-C8-BTNT regioisomers. (a) Single domain of **1a** after isolation. (b) Single domain of **2a** after isolation. (c)(e) Crystalline thin films composed of several single-crystalline domains fabricated by blade-coating for **2b** and **1b**, respectively. (d)(f) Single-crystal TFTs based on **2b** and **1b**, produced by isolation of single crystal domain from the films shown in (c) and (e), respectively. (a), (b), (d), and (f) are crossed-Nicols observations and (c) and (e) are non-polarized observations.

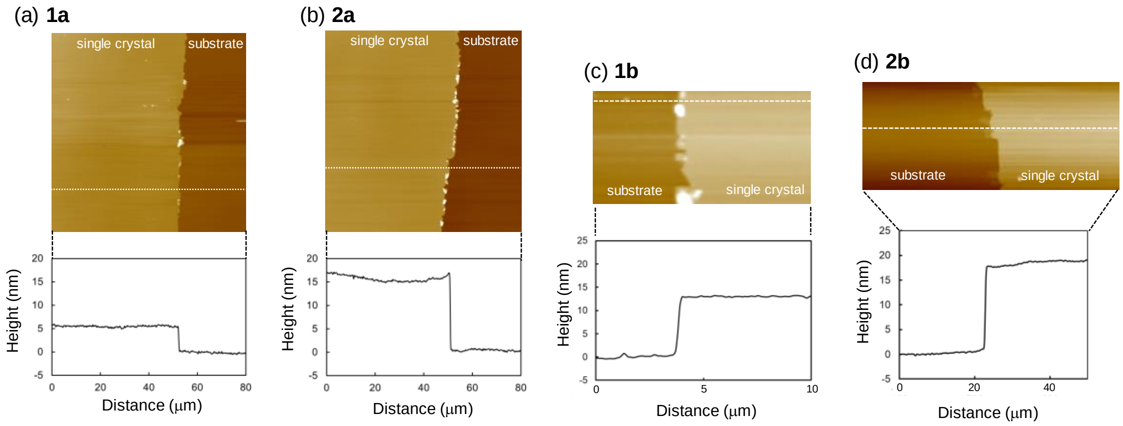

Figure S11. AFM images and their cross-sectional profiles of single crystalline thin films of (a) **1a**, (b) **2a**, (c) **1b**, and (d) **2b**.

6. References

S1. Niimi, K.; Kang, M. J.; Miyazaki, E.; Osaka, I.; Takimiya, K. *Org.Lett.* **2011**, *13*, 3430-3433.

S2. Mori, T.; Nishimura, T.; Yamamoto, T.; Doi, I.; Miyazaki, E.; Osaka, I.; Takimiya, K. *J. Am. Chem. Soc.* **2013**, *135*, 13900-13913.

S3. Messersmith, R. E.; Siegler, M. A.; Tovar, J. D. *Synlett* **2018**, *29*, 2499-2502.

S4. Abe, S.; Miyaura, N.; Suzuki, A. *Bull. Chem. Soc. Jpn.* **1992**, *65*, 2863-2865.

S5. Chen, Y. F.; Ma, R. Y.; Zhou, L. N.; Du, Z.; Zhang, T. *Heterocycles* **2016**, *92*, 1874-1881.

S6. Abe, M.; Mori, T.; Osaka, I.; Sugimoto, K.; Takimiya, K. *Chem. Mater.* **2015**, *27*, 5049-5067.

S7. Yamamura, Y.; Adachi, T.; Miyazawa, T.; Horiuchi, K.; Sumita, M.; Massalska-Arodź, M.; Urban, S.; Saito, K. *J. Phys. Chem. B* **2012**, *116*, 9255-9260.
